# The BCL-2 inhibitor APG-2575 resets tumor-associated macrophages toward the M1 phenotype, promoting a favorable response to anti-PD-1 therapy via NLRP3 activation

**DOI:** 10.1038/s41423-023-01112-y

**Published:** 2023-12-07

**Authors:** Fan Luo, Han Li, Wenjuan Ma, Jiaxin Cao, Qun Chen, Feiteng Lu, Miaozhen Qiu, Penghui Zhou, Zengfei Xia, Kangmei Zeng, Jianhua Zhan, Ting Zhou, Qiuyun Luo, Wentao Pan, Lin Zhang, Chaozhuo Lin, Yan Huang, Li Zhang, Dajun Yang, Hongyun Zhao

**Affiliations:** 1 State Key Laboratory of Oncology in South China, Guangdong Key Laboratory of Nasopharyngeal Carcinoma Diagnosis and Therapy, Guangdong Provincial Clinical Research Center for Cancer, Sun Yat-sen University Cancer Center, Guangzhou, China; 2 Ascentage Pharma (Suzhou) Co Ltd, 218 Xinghu Street, Suzhou, Jiangsu Province China

**Keywords:** BCL-2, APG-2575, ICIs, Macrophages, NLRP3, Cancer immunotherapy, Cancer microenvironment

## Abstract

The main challenges in the use of immune checkpoint inhibitors (ICIs) are ascribed to the immunosuppressive tumor microenvironment and the lack of sufficient infiltration of activated CD8+ T cells. Transforming the tumor microenvironment (TME) from “cold” to “hot” and thus more likely to potentiate the effects of ICIs is a promising strategy for cancer treatment. We found that the selective BCL-2 inhibitor APG-2575 can enhance the antitumor efficacy of anti-PD-1 therapy in syngeneic and humanized CD34+ mouse models. Using single-cell RNA sequencing, we found that APG-2575 polarized M2-like immunosuppressive macrophages toward the M1-like immunostimulatory phenotype with increased CCL5 and CXCL10 secretion, restoring T-cell function and promoting a favorable immunotherapy response. Mechanistically, we demonstrated that APG-2575 directly binds to NF-κB p65 to activate NLRP3 signaling, thereby mediating macrophage repolarization and the activation of proinflammatory caspases and subsequently increasing CCL5 and CXCL10 chemokine production. As a result, APG-2575-induced macrophage repolarization could remodel the tumor immune microenvironment, thus improving tumor immunosuppression and further enhancing antitumor T-cell immunity. Multiplex immunohistochemistry confirmed that patients with better immunotherapeutic efficacy had higher CD86, p-NF-κB p65 and NLRP3 levels, accompanied by lower CD206 expression on macrophages. Collectively, these data provide evidence that further study on APG-2575 in combination with immunotherapy for tumor treatment is required.

## Introduction

Immune checkpoint inhibitors (ICIs), which target programmed cell death protein-1 (PD-1) or its ligand PD-L1, have yielded durable responses and marked clinical benefits in a subset of patients with diverse cancers [1]. Unfortunately, a substantial proportion of cancer patients do not respond to immunotherapy, largely due to the complexity of cancer immunity [2]. For instance, when used alone, anti-PD-1/PD-L1 antibodies demonstrate clinical efficacy in only approximately 20% of non-small cell lung cancer (NSCLC) patients [3]. The heterogeneity of the tumor microenvironment (TME) leads to differences in the response to ICIs among cancer patients [4]. The majority of patients with immune “cold”, or “noninflamed”, tumors respond poorly to ICIs [5]. As such, there is an urgent need to find appropriate modalities for use in combination with ICIs to reverse the “cold” tumor state into a “hot” tumor state to increase antitumor activity.

Macrophages constitute the largest proportion of tumor-infiltrating immune cells and play a vital role in regulating the TME immune status and tumor progression [6–10]. M1-like tumor-associated macrophages (TAMs) are generally recognized as classically activated macrophages that are capable of mediating anticancer effects by directly killing tumor cells or by stimulating antitumor T-cell immunity [11, 12]. During cancer progression, TAMs can be polarized into the M2-like phenotype via cues within the TME [13–15]. M2-like TAMs are localized predominantly in the TME, where they inhibit T-cell functions and exhibit protumor activity by inducing immunosuppressive effects, including the production of inhibitory molecules [16], recruitment of immunosuppressive cells [17] and expression of T-cell immune checkpoint ligands [18, 19]. Thus, the repolarization of M2-like macrophages into M1-like macrophages is a promising strategy for enhancing T-cell antitumor immunity and ameliorating the immunosuppressive TME, thereby complementing existing antitumor immunotherapies.

The antiapoptotic protein BCL-2 plays crucial roles in regulating oncogenesis, tumor survival, lymphocyte development and the immune response [20]. Whether BCL-2 is pivotal to anticancer immunity, however, remains unclear. Venetoclax is a specific small molecule inhibitor of BCL-2 that can increase the number of PD-1 + CD8+ T effector memory cells and enhance the antitumor efficacy of ICIs [21], but the specific mechanism by which BCL-2 inhibition promotes antitumor T-cell immunity has not been identified. APG-2575 is a novel, orally bioavailable BH3 mimetic selective BCL-2 inhibitor with potent antitumor activity in various malignancies [22]. It was granted orphan drug designation by the U.S. Food and Drug Administration (FDA) for five indications: chronic lymphocytic leukemia (CLL), Waldenström macroglobulinemia (WN), multiple myeloma (MM), acute myeloid leukemia (AML), and follicular lymphoma (FL) (https://www.ascentagepharma.com/zwxw/). Several phase Ib/II clinical trials of APG-2575 for the treatment of hematological and solid tumors have been conducted worldwide [23, 24]. Our laboratory reported that APG-2575 exerts synthetic lethality with Bruton’s tyrosine kinase (BTK) and an MDM2-p53 inhibitor in the treatment of diffuse large B-cell lymphoma (DLBCL) [25]. Moreover, APG-2575 in combination with olverembatinib/HQP1351 [22] or homoharringtonine (HHT) [26] also showed synergistic antitumor effects in AML. However, little is known about whether APG-2575 potentiates the efficacy of ICIs.

In the present preclinical study, we observed that APG-2575 could be combined with ICIs to boost antitumor immunity in both humanized and syngeneic mouse tumor models. This effect is attributed to its ability to repolarize TAMs to the M1 phenotype through NLRP3 activation, which augments the secretion of the chemokines CCL5 and CXCL10 by M1 macrophages, thereby remodeling the tumor immune microenvironment from “cold” to “hot” and finally increasing the infiltration of activated CD8+ T cells. Collectively, our findings indicate that further clinical evaluation of APG-2575 and anti-PD-1 therapy as a combination strategy is warranted.

## Results

### APG-2575 potentiates the efficacy of anti-PD-1 therapy in humanized CD34+ and C57BL/6 mouse models

**Fig. 1:**
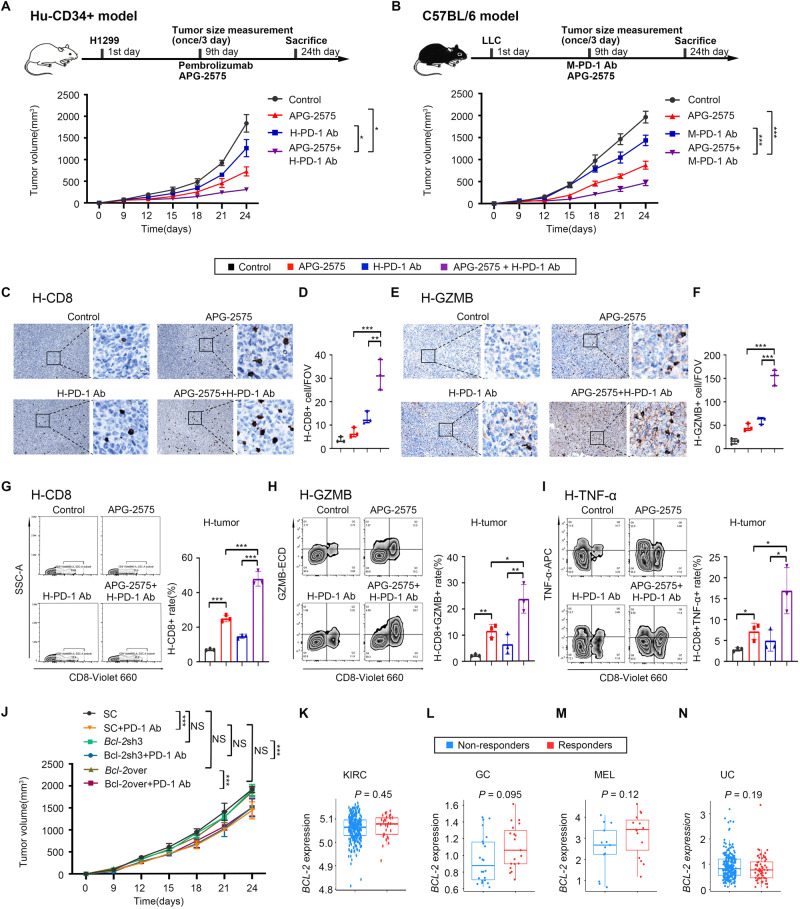
APG-2575 potentiates the efficacy of an immune checkpoint inhibitor in humanized CD34+ and C57BL/6 mouse models. **A**, **B** Tumor outgrowth in different groups, including the control, APG-2575, anti-PD-1 and combination treatment groups, in H1299 tumor-bearing humanized CD34+ model mice and in LLC tumor-bearing C57BL/6 mice. **C**–**F** Immunohistochemical analyses of CD8 and GZMB in hu-CD34+ mouse xenograft tumors. **G**–**I** Flow cytometric analysis of CD8+, CD8+GZMB+ and CD8+ TNF-α + T cells in hu-CD34+ mouse xenograft tumors. **J** Tumor outgrowth in *Bcl-2* knockdown and overexpression LLC tumor-bearing C57BL/6 mice in the different treatment groups. **K**–**N** Differences in *BCL-2* expression levels in various cancer types between nonresponders and responders who accepted immunotherapy. KIRC, kidney renal clear cell carcinoma, GC, gastric carcinoma; MEL, melanoma, UC, urothelial carcinoma

To explore whether APG-2575 increases the antitumor activity of PD-1 blockade, we utilized humanized mouse models in which human CD34+ HSCs were injected into the circulatory system of NSG mice. H1299 tumors in hu-CD34+ model mice were treated with control, APG-2575, PD-1 blockade or APG-2575+PD-1 blockade. APG-2575 and anti-PD-1 treatment both showed effective tumor inhibition; however, their effect was even greater in combination, dramatically suppressing tumor growth (Fig. 1A). Similarly, in C57BL/6 mice bearing LLC tumors, APG-2575 combined with anti-PD-1 treatment resulted in the most robust therapeutic response (Fig. 1B). These results suggest that APG-2575 combined with ICIs significantly enhances the therapeutic response in both human and murine tumor models.

CD8+ T cells mediate antitumor immunity. We investigated CD8+ T-cell function in hu-CD34+ mice. Immunohistochemistry (IHC) and flow cytometric analysis indicated that the numbers of tumor-infiltrating and splenic CD8+, Granzyme B+CD8+ and TNF-α + CD8+ T cells were higher in mice treated with APG-2575 + PD-1 blockade than in those treated with any single drug alone (Fig. 1C–I, Supplementary Fig. S1H–J). To confirm these results, we evaluated LLC tumors. Again, IHC and flow cytometric analysis indicated increases in CD8+, Granzyme B + CD8+ and TNF-α + CD8+ T cells in tumors and spleens in mice treated with APG-2575 + PD-1 blockade compared with those treated with APG-2575 or the anti-PD-1 antibody alone (Supplementary Fig. S1A–G, K–M).

We further examined the effect of APG-2575 on the CD8+ T-cell subpopulations of tumor-infiltrating lymphocytes (TILs) in LLC tumors. In response to APG-2575 treatment, we observed a reduction in naïve-like CD8+ T cells (Supplementary Fig. S1N) and enrichment of activated memory T cells, including effector memory T (TEM) cells and tissue-resident memory T (TRM) cells. The proportions of central memory T (TCM) cells remained unchanged (Supplementary Fig. S1O–Q). This increase in TEM-like cells was further enhanced upon combination treatment with the anti-PD-1 antibody and APG-2575. The overall gating strategy for T cells is shown in Supplementary Fig. S2. Collectively, these data show that APG-2575 and PD-1 blockade can synergistically increase the population of effector T cells in the TME and thus reduce tumor growth.

### The antitumor activity of APG-2575 is CD8+ T-cell driven

The above data warranted the exploration of whether the ability of APG-2575 to enhance the effect of PD-1 blockade on antitumor immunity might be the result of its suppressive effect on the target protein BCL-2, given that APG-2575 is a selective BCL-2 inhibitor. We first ectopically expressed *Bcl-2* and performed genetic knockdown of *Bcl-2* in LLC cells (Supplementary Fig. S6D). We then performed animal studies using the *Bcl-2* knockdown (KD), *Bcl-2* overexpression (OE) and scramble control LLC cell lines, which were subcutaneously injected into C57BL/6 mice. The mice were treated with saline or a mouse anti-PD-1 antibody. The mice treated with *Bcl-2* KD + PD-1 antibody or *Bcl-2* OE + PD-1 antibody exhibited tumor growth comparable to that in mice treated with only the mouse anti-PD-1 antibody, suggesting that genetic manipulation of *Bcl-2* had no effect on antitumor immunity (Fig. 1J). In addition, we performed analysis of data from various tumor tissues in the TCGA database to analyze the *BCL-2* expression level in patients who received immunotherapy and observed that responders to anti-PD-1 treatment had *BCL-2* expression levels comparable to those in nonresponders (Fig. 1K–N). These results further verified that there was no significant correlation between the tumor expression level of BCL-2 and the efficacy of PD-1 treatment. Therefore, the APG-2575-induced enhancement of the effects of anti-PD-1 therapy might not be associated with tumor BCL-2 inhibition.

**Fig. 2:**
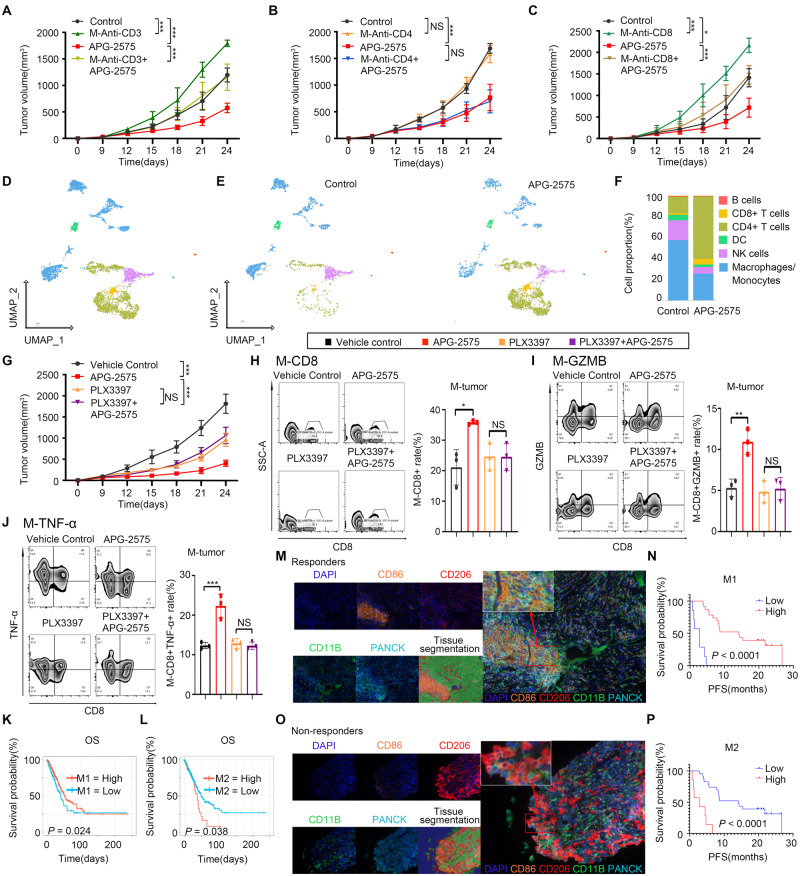
The antitumor activity of APG-2575 is CD8+ T-cell driven, and APG-2575 induces antitumor CD8+ T-cell immunity by regulating macrophages. **A**‒**C** Tumor volume comparison in APG-2575-treated LLC tumor-bearing C57BL/6 mice with or without depletion of CD3+, CD4+ or CD8+ T cells. **D** UMAP plot of human tumor-infiltrating CD45+ cells from the two groups merged and analyzed by scRNA-seq. **E** UMAP plots with annotated clusters of intratumoral immune cells from the control and APG-2575 groups. **F** The proportions of different immune cells in the control and APG-2575 groups. **G** Tumor volume comparison in APG-2575-treated LLC tumor-bearing C57BL/6 mice in the absence or presence of PLX3397. **H**‒**J** Flow cytometric analysis of CD8+, CD8 + GZMB+ and CD8+ TNF-α + T cells in C57BL/6 mouse xenograft tumors. **K**, **L** Kaplan‒Meier analysis of OS in patients in TCGA cohorts based on the M1 and M2 macrophage infiltration levels. **M**, **O** Representative multiplex immunofluorescence images demonstrating the CD11B + CD86+ (M1 macrophages), CD11B+CD206+ (M2 macrophages) and pan-CK (cancer cells) expression signatures in samples from responders and nonresponders. **N**, **P** Kaplan‒Meier analysis of PFS in patients based on M1 and M2 macrophage infiltration levels detected in tumors

We further examined whether the enhancement of anti-PD-1 activity may be the result of APG-2575-dependent cancer cell-intrinsic effects. We discovered that LLC and H1299 cells were resistant to APG-2575, with cytotoxic concentrations of up to 5 μM (Supplementary Fig. S3A). No reduction in cell viability or proliferation over 4 days of APG-2575 treatment was evident via real-time cell analysis (RTCA) (Supplementary Fig. S3B). Additionally, the results of the Annexin-V staining assay demonstrated that APG-2575 did not significantly induce either early apoptosis or late apoptosis in tumor cells (Supplementary Fig. S3C). Moreover, there were no observed alterations in immunomodulatory markers such as the expression of cell surface major histocompatibility complex class I (MHC-I) (Supplementary Fig. S3D) or PD-L1 (Supplementary Fig. S3E) following APG-2575 treatment. To investigate whether the antitumor efficacy of APG-2575 is immune dependent, we examined the response of T-cell-deficient nude mice bearing LLC or H1299 xenograft tumors to APG-2575 treatment. As expected, treatment with APG-2575 was ineffective in this mouse model (Supplementary Fig. S3F). We also used anti-CD3, anti-CD4 and anti-CD8 antibodies to eliminate CD3+, CD4+ or CD8+ T cells, respectively, in C57BL/6 mice and confirmed that depletion of CD3+ or CD8+ T cells was sufficient to abrogate the antitumor effect of APG-2575. In contrast, CD4+ T-cell elimination had no such effect, indicating the dispensable role of CD4+ T cells (Fig. 2A–C). These findings suggest that the antitumor effect of APG-2575 may be ascribed to its ability to activate CD8+ T-cell immunity.

To further explore whether APG-2575 directly promotes T-cell activation, we isolated splenic CD8+ T cells from C57BL/6 mice and then stimulated them with anti-CD3 and anti-CD28 antibodies in the presence or absence of APG-2575. APG-2575 had no promoting effect on CD8+ T-cell proliferation (Supplementary Fig. S3G). Splenic CD8+ T cells from APG-2575- and control-treated C57BL/6 mice were also isolated, and other T-cell-associated phenotypes were explored. Flow cytometric analysis identified comparable levels of T-cell apoptosis (Supplementary Fig. S3H), T-cell activation (CD69 and CD137) (Supplementary Fig. S3I, J) and T-cell exhaustion (PD-1 and CTLA-4) (Supplementary Fig. S3K–L) between cells from these mice. The above findings indicate that APG-2575 might not act directly on CD8+ T cells to enhance antitumor immunity.

### APG-2575 induced antitumor CD8+ T-cell immunity by regulating macrophages

To explore the potential mechanisms of APG-2575-induced antitumor T-cell immunity, human CD45+ immune cells were isolated from H1299 tumors growing in hu-CD34+ model mice in the control group and the APG-2575 treatment group for scRNA-seq analysis. After quality control and processing, all CD45+ immune cells were profiled and classified into 14 distinct clusters (Clusters 0-13), as shown in Supplementary Fig. S3M. Subsequently, we analyzed the expression patterns of known marker genes to define each cell cluster (Supplementary Table 5) and annotated six major immune cell subsets: B cells, CD4+ T cells, CD8+ T cells, dendritic cells (DCs), monocytes/macrophages (Mo/MFs), and natural killer (NK) cells (Fig. 2D). The dot plots that show the average scaled expression levels of selected marker genes for each cell cluster and each annotated cell type are presented in Supplementary Fig. S3N–O, respectively. Mo/MFs were the most abundant CD45+ subpopulation, followed by T cells. B cells were the least abundant population (Fig. 2D). APG-2575 primarily affected Mo/MFs and T cells among CD45+ immune cells. Following APG-2575 treatment, the proportion of Mo/MFs was markedly decreased, whereas both the CD4+ T-cell and CD8+ T-cell populations were markedly increased. APG-2575 administration might not alter the proportion of B cells in the TME, based on the results of scRNA-seq (Fig. 2E, F).

To further identify whether macrophages are involved in APG-2575-induced antitumor activity, mice bearing LLC tumors were treated with APG-2575 in the presence or absence of PLX3397, a colony-stimulating factor-1 receptor (CSF1R) inhibitor, which can deplete macrophages [27]. Macrophage depletion effectively disrupted the suppressive effect of APG-2575 on LLC tumors (Fig. 2G) and simultaneously abrogated APG-2575-induced antitumor activity and increased the numbers of CD8+ T cells (Fig. 2H), Granzyme B + CD8+ T cells (Fig. 2I) and TNF-α + CD8+ T cells (Fig. 2J). These data demonstrate that the antitumor activity and enhancing effect on CD8+ T-cell antitumor immunity of APG-2575 are macrophage dependent.

Correlation analysis based on TCGA data showed that M1 macrophages were positively correlated with CD8+ T cells (Supplementary Fig. S3P). Kaplan‒Meier analysis showed that patients with a high M1 infiltration level had significantly longer overall survival (OS) than those with a low M1 infiltration level (Fig. 2K, Supplementary Fig. S3Q). The opposite pattern was observed for M2 macrophages (Fig. 2L). We further validated the bioinformatic analysis results in NSCLC samples from patients who received immunotherapy by using a multiplex immunohistochemistry (mIHC) assay. We observed that patients with more M1 macrophage (CD11B+ CD86+) infiltration were more likely to have a better prognosis (Fig. 2M, N), while patients with more M2 macrophage (CD11B+ CD206+) infiltration had shorter survival times (Fig. 2O, P).

**Fig. 3:**
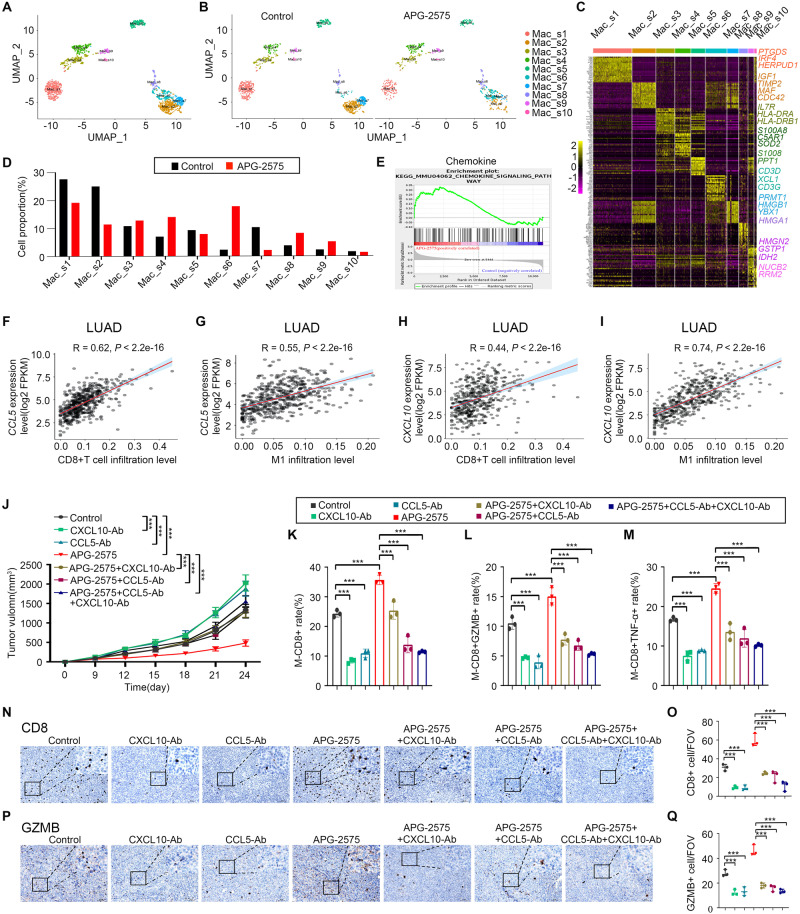
APG-2575 remodels the transcriptomic landscape of macrophages and CD8+ T cells and promotes CD8+ T-cell infiltration via the chemokines CCL5 and CXCL10. **A** UMAP plot from merged data of tumor-infiltrating Mo/MF populations. **B** UMAP plots with annotated clusters of Mo/MF cells from the control and APG-2575 groups. **C** A heatmap showing the differentially expressed genes (rows) among Mo/MF subpopulations (columns). Representative genes from each cluster are highlighted (right). **D** Ratios of the proportions of Mo/MF clusters across different regimens. **E** GSEA using genes differentially expressed between IL-4-activated RAW264.7 cells with control or APG-2575 treatment. **F**‒**I** Scatter plot showing the results of Pearson correlation analysis of *CCL5 and CXCL10 expression and* the infiltration of CD8+ T cells and M1 macrophages in TCGA cohorts. **J** Growth of LLC tumors in C57BL/6 mice treated with the indicated regimens. (**K**–**M**) Flow cytometric analysis of CD8+, CD8 + GZMB+ and CD8+ TNF-α+ T cells in C57BL/6 mouse xenograft tumors treated with the indicated regimens. **N**‒**Q** Immunohistochemical staining of CD8 and GZMB in C57BL/6 mouse xenograft tumors

Furthermore, reclustering of the Mo/MF populations derived from the scRNA-seq data identified ten subpopulations (Fig. 3A, C). Cells in the Mac_s3, Mac_s6, Mac_s8 and Mac_s9 subpopulations displayed significant upregulation of proinflammatory genes (e.g., *HLA*‒*DRA*, *HLA*‒*DRB1*, *IL1β*, *XCL1*, *CD3G*, *HMGA1*, *HMGN2*, *GSTP1* and *IDH2*). Cells in the Mac_s1, Mac_s2, Mac_s5, Mac_s7 and Mac_s10 subpopulations exhibited upregulation of anti-inflammatory genes (e.g., *PTGDS*, *IRF4*, *HERPUD1*, *IGF1*, *MAF*, *CDC42* and *PRMT1*). Cells in the Mac_s4 subpopulation highly expressed *SPP1*, *VCAN*, and *MMP9*, suggesting that they may be M0 macrophages [28–30]. APG-2575 treatment significantly reduced the Mac_s1, Mac_s2 and Mac_s7 subpopulations and increased the calculated Mac_s3, Mac_s6, Mac_s8 and Mac_s9 subpopulations (Fig. 3B, D).

Taken together, these results show that APG-2575 reshapes the transcriptomic landscape of immune cells, which attenuates M2-like macrophage signatures and enhances M1-like macrophage signatures and that cytotoxic T cells mostly alleviate immunosuppression in the TME.

### APG-2575 promoted CD8+ T-cell infiltration via the chemokines CCL5 and CXCL10

The mechanism by which APG-2575 increases CD8+ T-cell infiltration into the TME was next investigated. Based on the RNA-seq results in IL-4-activated RAW264.7 cells after control or APG-2575 treatment, a series of chemokines, including *Ccl3*, *Ccl4*, *Ccl5*, *Ccl7*, *Cxcl2*, and *Cxcl10*, were upregulated after APG-2575 treatment. GSEA also revealed that the gene signature CHEMOKINE_SIGNALING PATHWAY was enriched in both treatment groups (Fig. 3E, Supplementary Fig. S4A). We first confirmed whether APG-2575 changed the mRNA expression levels of these chemokines in macrophages. *Ccl5* and *Cxcl10* mRNA levels were increased considerably in the presence of APG-2575 (Supplementary Fig. S4B–D). APG-2575 also enhanced the secretion of CCL5 and CXCL10 in the supernatants of IL-4-activated macrophages, an effect that was blocked by inhibition of both NF-κB and NLRP3 (Supplementary Fig. S4E–H).

Importantly, we used the TIMER 2.0 database and discovered that the expression of *CCL5* and *CXCL10* showed positive relationships with the infiltration levels of CD8+ T cells and M1 macrophages (Fig. 3F–I). Using an in vitro Transwell migration assay, the migration of activated CD8+ T cells toward supernatants of APG-2575-treated IL-4-activated macrophages was significantly elevated compared with that toward IL-4-activated macrophages in the control treatment group (Supplementary Fig. S4I, J). Depletion of CCL5 and CXCL10 led to a reduction in CD8+ T-cell migration in vitro. A more obvious reduction in CD8+ T-cell migration was observed when CCL5 and CXCL10 were simultaneously depleted (Supplementary Fig. S4I, J). To address the role of CCL5 and CXCL10 in CD8+ T-cell trafficking in vivo, C57BL/6 mice were inoculated with LLC cells and injected intraperitoneally with anti-CCL5 and anti-CXCL10 antibodies or isotype control. Compared with the control treatment, CCL5 and CXCL10 blockade abrogated the effect of APG-2575 on tumor growth (Fig. 3J). We found that blocking CCL5 or CXCL10 in APG-2575-treated mice reduced CD8+, Granzyme B + CD8+ and TNF-α + CD8+ T-cell infiltration to the same level observed in control-treated mice (Fig. 3K–Q). These results suggest that the APG-2575-induced increase in CD8+ T-cell infiltration into the TME is mediated through increased production of CCL5 and CXCL10.

### APG-2575 effectively repolarized M2-like macrophages to the M1 phenotype

**Fig. 4:**
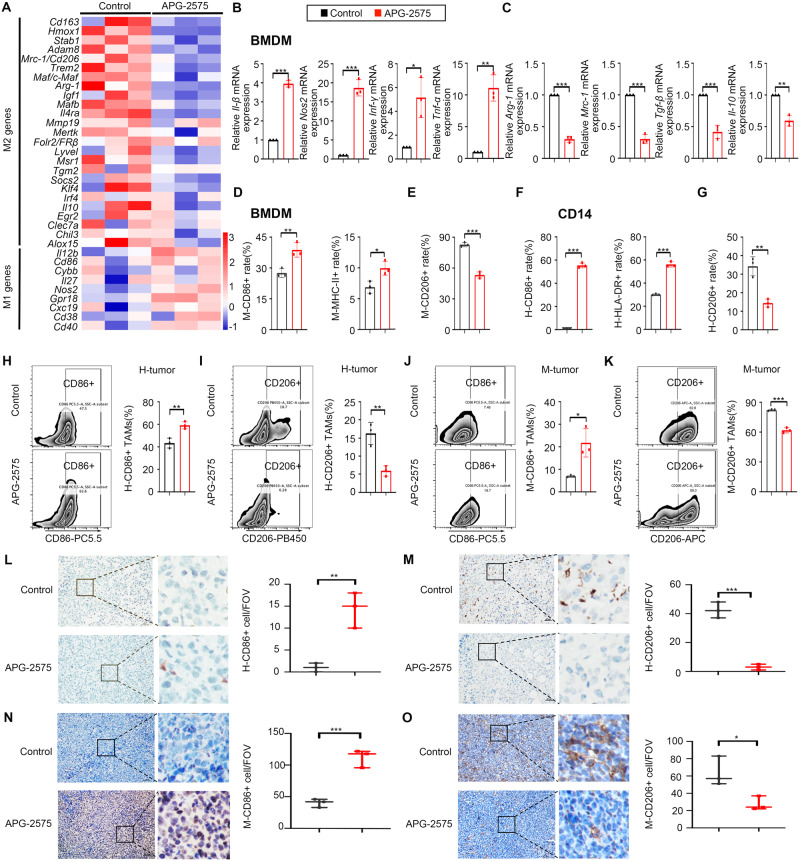
APG-2575 effectively repolarized M2-like macrophages to the M1 phenotype. **A** A heatmap of the normalized mean-centered mRNA expression levels of genes associated with M1 or M2 macrophages in the control and APG-2575 treatment groups. **B**, **C** IL-4-activated BMDMs were treated with or without APG-2575. The mRNA expression of M1/M2-related markers was analyzed. **D**, **E** Quantification of CD86, MHC-II and CD206 expression in IL-4-activated BMDMs with or without APG-2575 treatment. **F**, **G** Quantification of CD86, HLA-DR and CD206 expression in IL-4-activated CD14+ monocyte-derived macrophages with or without APG-2575 treatment. **H**–**K** Flow cytometric analysis of CD86 and CD206 in TAMs from hu-CD34+ and C57BL/6 mice treated with PBS or APG-2575. **L**, **M** Immunohistochemical staining of CD86 and CD206 in humanized CD34+ mouse xenograft tumors. **N**, **O** Immunohistochemical staining of CD86 and CD206 in C57BL/6 mouse xenograft tumors

To understand the regulatory effect of APG-2575 on macrophages, we isolated tumor-associated macrophages (TAMs) from LLC tumors and profiled the global transcriptome using RNA sequencing. TAMs from the control group displayed a transcriptional profile characteristic of M2 macrophages. Among the downregulated genes, genes such as *Cd163*, *Cd206* and *Il-10* were expressed preferentially in M2-like macrophages in the APG-2575 group compared with the control group. In contrast, known M1-related gene markers, such as *Gpr18*, *Nos2* and *Cd86*, were significantly upregulated in the APG-2575 group compared with the control group (Fig. 4A).

To demonstrate whether APG-2575 directly regulates macrophage polarization, we examined mRNA and protein expression in both murine BMDMs and human blood-derived CD14+ monocyte-derived macrophages stimulated in vitro with an anti-inflammatory cytokine (IL-4). Compared with control treatment, APG-2575 treatment significantly upregulated the mRNA expression of *IL-1β*, *NOS2*, *IFN-γ* and *TNF-α* (Fig. 4B, Supplementary Fig. S5A) and the costimulatory molecules CD86, MHC-II and HLA-DR (M1-related markers) (Fig. 4D, F) but reduced the mRNA expression of *ARG-1*, *MRC-1*, *TGF-β* and *IL-10* (Fig. 4C, Supplementary Fig. S5B) and of CD206 (M2-related marker) (Fig. 4E, G). These results were confirmed using APG-2575 in IL-4-activated RAW264.7 (Supplementary Fig. S5C–F) and IL-4-activated THP-1-derived macrophages (Supplementary Fig. S5G, H). These data indicate that the effect of APG-2575 on the polarization of M2-like macrophages toward the M1-like phenotype is universal. When we cocultured APG-2575-treated cancer cells with IL-4-activated macrophages, APG-2575-treated cancer cells did not alter the expression of CD86 and CD206 (Supplementary Fig. S5I). This suggests that APG-2575-treated cancer cells have no impact on M1 macrophage polarization.

Additionally, in hu-CD34+ and C57BL/6 mice, APG-2575 treatment consistently elevated the level of CD86 while reducing that of CD206 in tumor-infiltrating macrophages (Fig. 4H–K), as determined by flow cytometry, accompanied by upregulation of CD86 and downregulation of CD206 in tumor tissues, as detected by IHC (Fig. 4L–O). These data confirm that APG-2575 controls the switch in macrophage polarization between immunostimulatory and inhibitory. Notably, there was no significant difference in the proportion of human CD68 + CD11B+ or murine F4/80 + CD11B+ total macrophages, based on flow cytometric analysis of tumor tissues in both the humanized CD34+ and C57BL/6 mouse models, suggesting that APG-2575 does not influence the overall infiltration of macrophages (Supplementary Fig. S5J).

Previous studies have reported that macrophage migration to the tumor microenvironment is a critical event in tumor development [13, 31]. We next determined whether APG-2575 induces M2 macrophage migration using a Transwell assay. We observed that the extent of migration induced by APG-2575 was similar to that induced by the control treatment (Supplementary Fig. S5K–L). To further explore whether APG-2575 can induce a decrease in the population of M2 macrophages by promoting macrophage apoptosis, we treated macrophages with LPS + IFN-γ, which induces M1 macrophage polarization, and IL-4, which induces M2 macrophage polarization, as well as with conditioned medium (CM) from different tumor cells, which induces differentiation into TAMs. Cell death, as assessed by Annexin V/PI staining and flow cytometry, was not increased in M1 macrophages, M2 macrophages or TAMs after APG-2575 treatment (Supplementary Fig. S5M–O). Therefore, these results suggest that APG-2575 does not influence macrophage infiltration and that its regulatory effect on macrophages may primarily involve a switch in polarization from the M2 to the M1 phenotype.

### APG-2575 resets M2-like macrophages toward the M1 phenotype by activating NLRP3

**Fig. 5:**
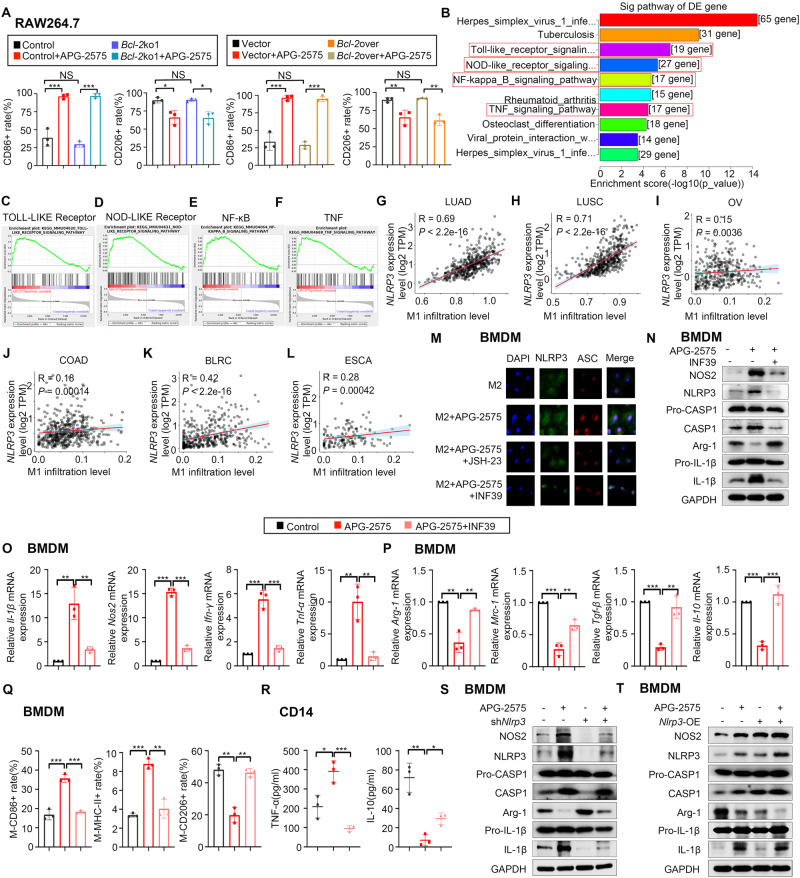
APG-2575 enhanced M1 polarization by upregulating NLRP3 expression. **A** Quantification of CD86 and CD206 expression in IL-4-activated *Bcl-2*-knockout or *Bcl-2*--overexpressing RAW264.7 cells treated with APG-2575 or control. **B**–**F** KEGG pathway analysis and GSEA using genes differentially expressed between IL-4-activated RAW264.7 cells with the control or APG-2575 treatment. **G**–**L** Scatter plot showing the results of Pearson correlation analysis between the estimated M1 macrophage infiltration score and *NLRP3* gene expression level in various cancer types. **M** Representative immunofluorescence staining of NLRP3, ASC, and DAPI in IL-4-activated BMDMs after treatment with APG-2575 in combination with JSH-23 or INF39. Scale bar, 20 μm. **N** Western blot analysis of NOS2, NLRP3, caspase-1, Arg-1, and IL-1β in IL-4-activated BMDMs cultured with APG-2575 in the presence or absence of INF39. **O**, **P** The mRNA expression levels of M1/M2-related markers in IL-4-activated BMDMs treated with APG-2575 in the presence or absence of INF39. **Q** Quantification of CD86, MHC-II and CD206 expression in IL-4-activated BMDMs treated with APG-2575 in the presence or absence of INF39. **R** IL-4-activated CD14+ monocyte-derived macrophages treated with or without APG-2575. The TNF-α concentration in the supernatants was measured by a CBA, and the IL-10 concentration in the supernatants was measured by ELISA. **S**, **T** Western blot analysis of NOS2, NLRP3, caspase-1, Arg-1, and IL-1β in IL-4-activated BMDMs with stable *Nlrp3* knockdown and overexpression and treated with APG-2575 or control. LUAD, lung adenocarcinoma, LUSC, lung squamous cell carcinoma, OV, ovarian serous cystadenocarcinoma, COAD, colon adenocarcinoma; BLCA, bladder urothelial carcinoma, ESCA, esophageal carcinoma

To gain insight into the underlying mechanism through which APG-2575 resets M2-like macrophages toward the M1 phenotype, we first determined whether BCL-2 plays a key role in APG-2575-induced M1 polarization. We established *BCL-2* knockout and overexpression RAW264.7 and THP-1 cell lines (Supplementary Fig. S6A, B). Neither *BCL-2* knockout nor *BCL-2* overexpression in macrophages influenced APG-2575-induced M1 polarization (Fig. 5A, Supplementary Fig. S5C). We also established murine LLC cell lines with stable *Bcl-2* knockdown and overexpression and human H1299 cell lines with stable *BCL-2* knockdown by RNAi and overexpression (Supplementary Fig. S5D, E). When we cocultured these APG-2575-treated cancer cells with genetic alteration of *BCL-2* with IL-4-activated macrophages, we found that neither downregulation nor upregulation of *BCL-2* in the cancer cells altered the expression of M1/M2-related markers (Supplementary Fig. S5F, G). The above data indicate that genetic alteration of *BCL-2* in macrophages or cancer cells has no effect on APG-2575-induced M1 macrophage polarization.

We performed RNA-seq in IL-4-activated RAW264.7 cells after control or APG-2575 treatment. KEGG pathway analysis and GSEA indicated that the APG-2575-regulated pathways were enriched in several subsystems, including Toll-like receptor (TLR) signaling, NOD-like receptor signaling, NF-κB signaling and TNF signaling pathways (Fig. 5B–F). To further investigate the molecular mechanism through which APG-2575 resets TAMs, we initially focused on the downstream proteins of TLRs, such as MyD88, TRIF, and TRAF6, as well as three members of the mitogen-activated protein kinase (MAPK) family, namely, JNK, Erk1/2, and p38, which are key regulators of proinflammatory factors [32]. We found that the expression of MyD88, TRIF and TRAF6 remained unchanged and that the MAPK proteins were not phosphorylated in M2 macrophages after APG-2575 treatment (Supplementary Fig. S6H), implying that TLR-MyD88-TRIF signaling and MAPK pathways might not be involved in the APG-2575-induced macrophage phenotype switch.

Notably, members of the NOD-like receptor (NLR) family have been shown to contribute to macrophage polarization, for example, NLRP3 [33], NLRC4 [34], and NLRP7 [35]. Therefore, we also examined the expression levels of these three NLRs in macrophages treated with APG-2575 and found that the NLRP3 expression level was significantly elevated compared to that in untreated control cells, while the expression of both NLRC4 and NLRP7 remained unaltered (Supplementary Fig. S6H). In addition, activation of NLRP3 has been reported to mediate M1 polarization [33] and induce proinflammatory mediators, including IL-1β, TNF-α, CXCL9 and CXCL10 [36], as demonstrated by qRT‒PCR (Supplementary Fig. S6I–K). Moreover, activation of NF-κB, which was activated in APG-2575-treated macrophages, is required for the transcription of pro-IL-1β and NLRP3 [37]. Correlation analysis also showed a positive correlation between the NLRP3 gene expression leve and the M1 macrophage infiltration level in various cancer types, based on analyses of TCGA data (Fig. 5G–L).

We then explored whether the expression of NLRP3, which is associated with M1 polarization, was involved in the response to APG-2575 treatment. The immunofluorescence staining assay suggested that NLRP3 expression was increased and that NLRP3 was colocalized with ASC in APG-2575-treated IL-4-activated BMDMs, and this effect was attenuated by treatment with INF39, an inhibitor of NLRP3 (Fig. 5M). Moreover, APG-2575 increased the expression of caspase-1, IL-1β, and NLRP3. INF39 treatment reduced the expression of IL-1β, caspase-1, and NOS2 expression and increased the expression of Arg-1 induced by APG-2575 treatment (Fig. 5N, Supplementary Fig. S6L). Consistently, APG-2575 induced the mRNA expression of M1-related markers and reduced the mRNA expression of M2-related markers, which was also be impeded by INF39 (Fig. 5O, P, Supplementary Fig. S7A–D). INF39 also markedly suppressed the APG-2575-induced changes in the expression of repolarization-related markers, as measured by flow cytometry (Fig. 5Q, Supplementary Fig. S7E–G). Furthermore, the biological processes of the reduction in the IL-10 concentration and increase in the TNF-α concentration in the supernatant observed after APG-2575 treatment were abrogated by INF39 treatment (Fig. 5R, Supplementary Fig. S7H–K).

Most importantly, we established BMDMs with stable *Nlrp3* knockdown and overexpression (Supplementary Fig. S7L) and observed that the APG-2575-induced increases in NLRP3, IL-1β and caspase-1 expression were further enhanced by overexpression of *Nlrp3*. However, knockdown of *Nlrp3* attenuated the expression of these proteins (Fig. 5S, T). Consistently, flow cytometric analyses demonstrated that *Nlrp3* knockdown blocked APG-2575-induced CD86 upregulation and CD206 downregulation, while *Nlrp3* overexpression, conversely, enhanced APG-2575-induced CD86 upregulation and CD206 downregulation (Supplementary Fig. S7M–P). Taken together, these findings indicate that APG-2575 promotes M1 macrophage polarization through an increase in NLRP3 expression.

### APG-2575 induced NLRP3 transcription by enhancing NF-κB nuclear localization

**Fig. 6:**
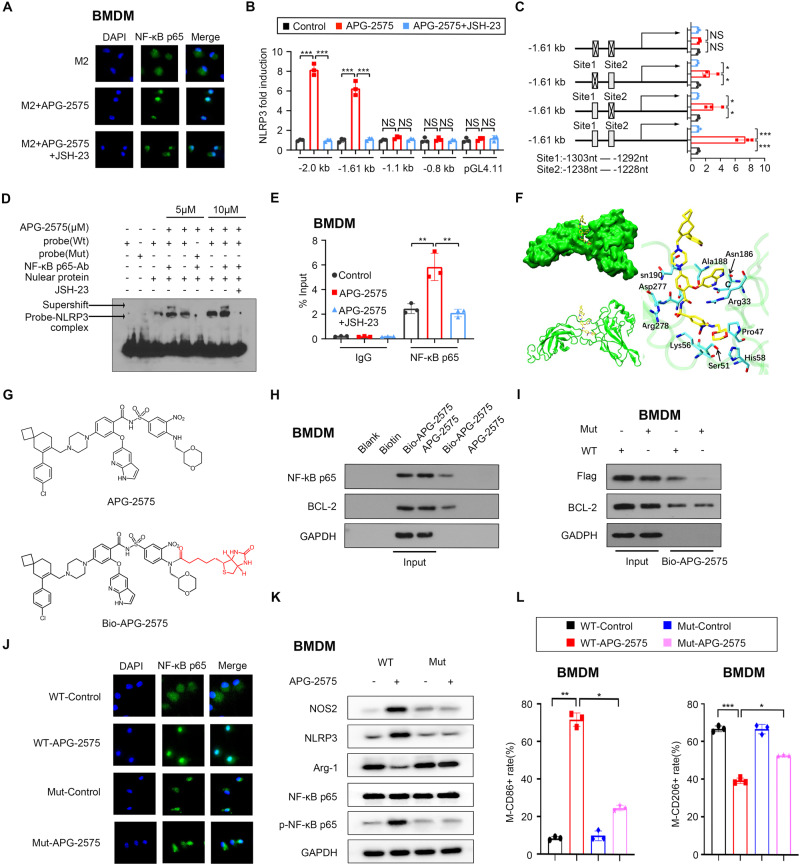
APG-2575 induced NLRP3 transcription by enhancing the nuclear localization of NF-κB. **A** NF-κB p65 localization in IL-4-activated BMDMs with or without APG-2575 treatment was examined using a confocal fluorescence microscope. Green, NF-κB p65; blue, DAPI. Scale bar, 20 μm. **B** Luciferase reporter assays with distinct *Nlrp3* reporters in Raw264.7 cells activated by IL-4 and then treated with APG-2575 or control. **C** Luciferase reporter assays with different versions of the 1.61 kb *Nlrp3* reporters in RAW264.7 cells activated by IL-4 and then treated with APG-2575 or control. **D** EMSAs were performed in nuclear extracts with a biotin-labeled NF-κB probe (containing the NF-κB consensus binding sequence). IL-4-activated RAW264.7 cells were treated with APG-2575 or control. **E** ChIP assay showing the recruitment of NF-κB p65 to the Nlrp3 promoter in IL-4-activated BMDMs. **F** The simulated complex structure and binding mode of APG-2575 with the RELA protein. **G** Chemical structures of APG-2575 and biotin-labeled APG-2575 (Bio-APG-2575). **H** Bio-APG-2575 was added to streptavidin-agarose beads, and the mixture was incubated. Biotin alone was used as a control. Lysates were prepared from BMDMs. **I** BMDMs were transfected with WT (wild type) NF-κB p65 or mutant NF-κB p65 (Arg33A/Lys56A/Asp277A/Arg278A). Lysates were used for pulldown assays to detect APG-2575 binding using the pulldown assay procedure described in (**H**). **J** NF-κB p65 localization in IL-4-activated BMDMs transfected with WT NF-κB p65 or mutant NF-κB p65 and treated with or without APG-2575 was examined using a confocal fluorescence microscope. Green, NF-κB p65; blue, DAPI. Scale bar, 20 μm. **K** Western blot analysis of NOS2, NLRP3, Arg-1, and NF-κB p65 in IL-4-activated BMDMs transfected with WT NF-κB p65 or mutant NF-κB p65 and treated with or without APG-2575. **L** Flow cytometric analysis of CD86 and CD206 in IL-4-activated BMDMs transfected with WT NF-κB p65 or mutant NF-κB p65 and treated with or without APG-2575

We further sought to identify the effect of APG-2575 that contributes to NLRP3 activation. A recent study demonstrated that NF-κB-mediated NLRP3 activation in macrophages was induced by the potent M1 macrophage inducer LPS [38]. Here, we assessed the effects of APG-2575-mediated activation on NF-κB signaling. APG-2575 treatment resulted in the entry of NF-κB p65 into the nucleus of IL-4-activated BMDMs (Fig. 6A) and IL-4-activated RAW264.7 cells (Supplementary Fig. S8A). APG-2575 reduced the cytoplasmic NF-κB p65 protein level and increased the nuclear NF-κB p65 protein level (Supplementary Fig. S8B, C). We then evaluated the effects of NF-κB pathway involvement using the NF-κB pathway inhibitor JSH-23. As shown in Fig. 6A and Supplementary Fig. S8A, JSH-23 inhibited the APG-2575-induced increase in the nuclear entry of NF-κB p65. When we used JSH-23 to treat IL-4-activated macrophages, we found that JSH-23 significantly abrogated the APG-2575-induced increases in the levels of M1-related mRNA markers (Supplementary Fig. S8D, F, H) and the secretion of the M1-associated cytokine TNF-α (Supplementary Fig. S8J, K). Simultaneously, JSH-23 suppressed the APG-2575-induced decreases in the levels of M2-associated markers (Supplementary Fig. S8E, G, I) and secretion of the M2-associated cytokine IL-10 (Supplementary Fig. S8L–O). JSH-23 also markedly attenuated the APG-2575-induced repolarization of M2 macrophages to M1 macrophages (Supplementary Fig. S9A–D). As anticipated, the protein levels of p-NF-κB p65 and NLRP3 were decreased upon JSH-23 treatment in IL-4-activated macrophages treated with APG-2575, accompanied by downregulation of caspase-1 and IL-1β expression (Supplementary Fig. S9E). These results indicate that activation of the NF-κB pathway is a pivotal event in APG-2575-induced macrophage polarization.

Next, a series of assays were conducted to investigate whether APG-2575 transcriptionally regulates NLPR3. We researched the potential role of NF-κB p65 in the regulation of NLRP3 because the NF-κB signaling pathway was activated after APG-2575 treatment, as shown in the above RNA-seq analysis (Fig. 5B, E). We performed luciferase reporter assays with both HEK293 and RAW264.7 cells, finding that APG-2575 enhanced the transcriptional activity of NF-κB p65 and NLPR3 (Supplementary Fig. S9F, G). Additionally, promoter analysis of NLRP3 demonstrated that a 900-bp region (−2000 to −1100 bp) was responsible for the elevated activity of APG-2575 (Fig. 6B). In silico prediction identified two NF-κB p65 binding sites within this 900-bp region, which were validated in a previous study. Mutation of site 1 or site 2 alone attenuated but failed to abolish the promoting effects of APG-2575 on transcriptional activity (Fig. 6C). Combined mutation of both sites 1 and 2 abolished the activating effect of APG-2575 (Fig. 6C). The EMSA results further confirmed that APG-2575 promoted the direct binding of the NF-κB p65 probe to the NLRP3 promoter (Fig. 6D). By performing ChIP assays, we showed that APG-2575 promoted the recruitment of NF-κB p65 to the NLRP3 promoter, thus promoting NLRP3 expression (Fig. 6E, Supplementary Fig. S9H). These data elucidate that NF-κB p65 binding sites are responsible for APG-2575-mediated activation of NLRP3. We validated this finding by establishing mouse models. Both JSH-23 and INF39 weakened the antitumor efficacy of APG-2575 (Supplementary Fig. S9I, J). Flow cytometric and immunohistochemical staining analyses of TAMs revealed that JSH-23 and INF39 significantly impaired the APG-2575-induced decrease in M2 macrophages and increase in M1 macrophages (Supplementary Figs. S9K–N, S10A–H). The above data indicate that APG-2575 enhances M1 polarization in vivo by activating NF-κB/NLRP3 signaling.

### Binding modes of APG-2575 to the NF-κB p65 protein

We also explored whether APG-2575-activated NF-κB/NLRP3 signaling relies on the regulation of its target BCL-2. *BCL-2* knockout or overexpression in macrophages did not influence the APG-2575-mediated activation of the NF-κB/NLRP3 signaling pathway (Supplementary Fig. S10I–K, N–P). We further cocultured APG-2575-treated tumor cells with *BCL-2* knockdown or overexpression with IL-4-activated macrophages and measured the protein levels of p-NF-κB p65 and NLRP3 in IL-4-activated macrophages, and we obtained consistent results (Supplementary Fig. S10L–M, Q–R), indicating that genetic alteration of *BCL-2* expression in cancer cells also had no impact on APG-2575-mediated activation of NF-κB/NLRP3 signaling. Hence, the regulation of the NF-κB/NLRP3 pathway by APG-2575 might be independent of its target BCL-2.

To identify the physical site at which the NF-κB p65 protein and APG-2575 interact, we conducted a molecular docking and simulation study using the crystal structure of RELA (NF-κB p65) (PDB: 1IKN). The binding mode of mouse RELA with APG-2575 is illustrated in Supplementary Fig. S11A. APG-2575 formed suitable steric complementarity with the binding site formed by Ser276, Met32, Asp277, Arg33, Arg273, Arg278 and Asp277. A more negative score (−9.313) was obtained for the APG-2575-RELA interaction, suggesting that APG-2575 has high binding affinity for RELA. To predict the binding site of APG-2575 in RELA, a per-residue decomposition energy calculation was performed. The lowest binding free energy for APG-2575 with the RELA protein was computed to be −59.33 kcal/mol for pose7, as shown in Supplementary Fig. S11B. The per-residue energy decomposition (PRED) values of residues within 4.0 Å between APG-2575 and the RELA protein for all poses and the final stable complex are shown in Supplementary Fig. S11C, D. The most stable simulated complex structure and binding mode of APG-2575 and the RELA protein are shown in Fig. 6F. Only the residues near the interaction site with energy contributions of greater than 1.00 kcal/mol are displayed in the binding mode visualization. According to energy decomposition analysis, Arg33, Lys56, Asp277, and Arg278 appear to function as the major interacting residues, playing a crucial role in stabilizing the complex of APG-2575 and the RELA protein.

We used biotin-labeled APG-2575 (**Bio-APG-2575**; Fig. 6G) and then determined whether APG-2575 binds to NF-κB p65 in cell lysates using a biotinylated protein interaction pulldown assay. Bio-APG-2575 was added to streptavidin-agarose beads, and lysates from BMDMs and RAW264.7 macrophages were added to the mixture. Our results indicate that Bio-APG-2575 binds to the NF-κB p65 protein in lysates of both BMDMs and RAW264.7 macrophages (Fig. 6H, Supplementary Fig. S11E). These results further confirm that APG-2575 directly binds NF-κB p65. As expected, probing for the BCL-2 protein, known to be the target of APG-2575, also showed an interaction with APG-2575 (Fig. 6H, Supplementary Fig. S11E).

According to the results of the molecular docking and simulation studies, Arg33, Lys56, Asp277 and Arg278 appeared to be key residues, based on their energy values. We then mutated Arg33/Lys56/Asp277/Arg278 to Ala to confirm their involvement in the APG-2575–NF-κB p65 interaction. Two plasmids, PCDNA3.1-(mouse, WT)-flag and PCDNA3.1-NF-κB p65 (mouse, Arg33A/Lys56A/Asp277A/Arg278A)-flag, were constructed and transfected into BMDMs and RAW264.7 macrophages. We determined whether Bio-APG-2575 binds to mutated NF-κB p65 in transfected BMDMs using biotinylated APG-2575 pulldown assays. Our results showed that Bio-APG-2575 bound to wild-type NF-κB p65 in BMDM and RAW264.7 macrophage lysates, while significantly reduced binding to mutant NF-κB p65 was observed in lysates of BMDMs and RAW264.7 macrophages (Fig. 6I, Supplementary Fig. S11F). These results suggest that Arg33, Lys56, Asp277 and Arg278 participate in the APG-2575–NF-κB p65 interaction.

More importantly, we observed increased entry of NF-κB p65 into the nucleus and increased expression of NLRP3 upon APG-2575 treatment in IL-4-activated macrophages, and these effects were abolished by the NF-κB p65 mutation, as evidenced by immunofluorescence staining assays (Fig. 6J, Supplementary Fig. S11G, H). Moreover, the NF-κB p65 mutation resulted in attenuation of APG-2575-induced NLRP3 expression (Fig. 6K, Supplementary Fig. S11I) and abrogation of APG-2575-mediated polarization of M2 macrophages to M1 macrophages (Fig. 6L, Supplementary Fig. S11J), as evidenced by western blotting and flow cytometry.

### The NF-κB/NLRP3 signaling pathway is positively related to the infiltration of M1-like macrophages and the efficacy of immunotherapy in patients

**Fig. 7:**
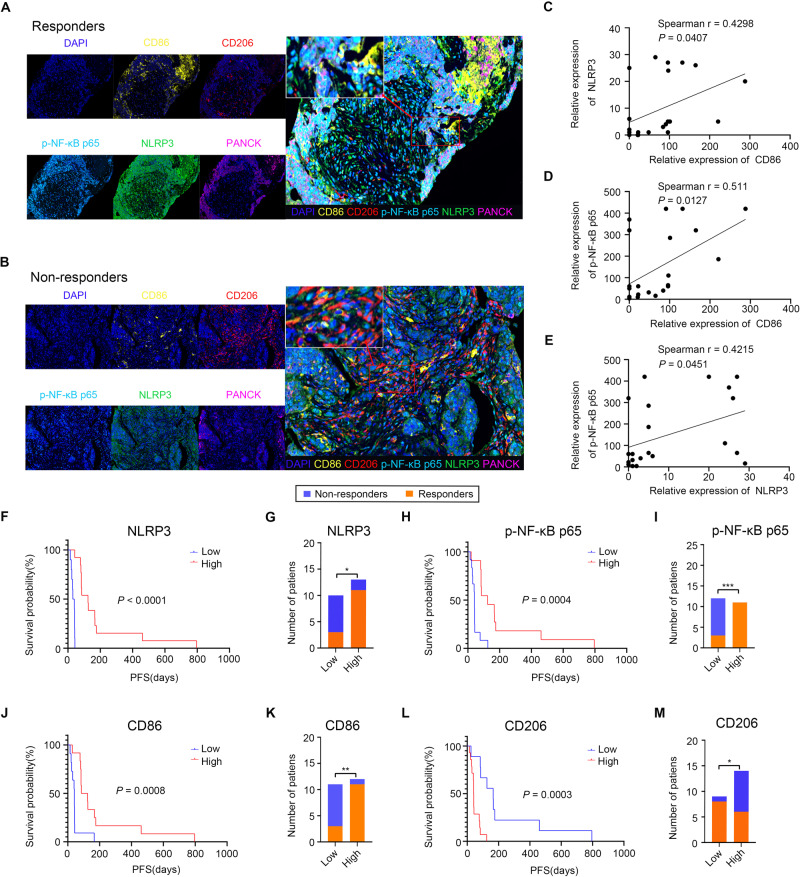
NF-κB/NLRP3 signaling pathway activity is positively related to M1-like TAM infiltration levels as well as the efficacy of an immune checkpoint inhibitor in NSCLC. **A**, **B** Representative multiplex immunofluorescence images demonstrating the protein expression of CD86, CD206, p-NF-κB p65, NLRP3 and pan-CK in samples from responders and nonresponders. **C**–**E** Statistical charts showing the correlations between CD86 and NLRP3, CD86 and p-NF-κB p65, and NLRP3 and p-NF-κB p65 expression. **F**, **H**, **J**, **L** Kaplan‒Meier analysis of PFS in patients based on the level of NLRP3, p-NF-κB p65, CD86 or CD206 on macrophages detected in tumors. **G**, **I**, **K**, **M** Correlation analysis showed that NLRP3, p-NF-κB p65, CD86 and CD206 on macrophages were significantly associated with the immune response

**Fig. 8:**
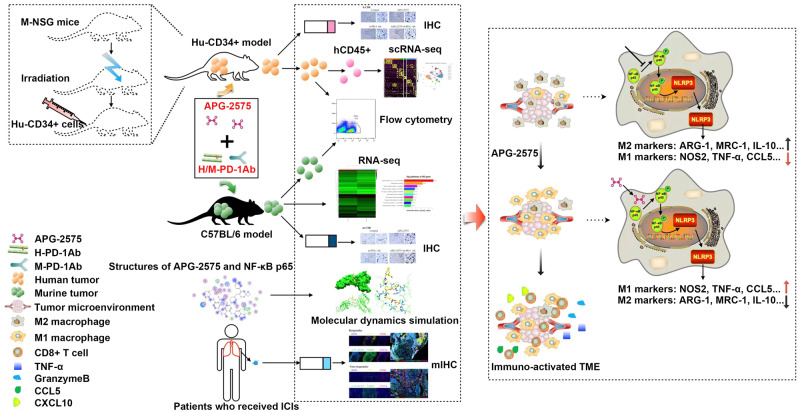
Graphical summary of the results. APG-2575 can synergize with ICIs through a mechanism involving the repolarization of TAMs from the M2 to the M1 phenotype, further enhancing CD8+ T-cell recruitment into the TME via the augmentation of CCL5 and CXCL10 secretion and thereby improving tumor immunosuppression

We performed mIHC assays to study the associations between M1/M2 macrophage numbers and p-NF-κB p65 and NLRP3 levels in the tumor tissues of NSCLC patients who received immunotherapy. The detailed clinical characteristics of all the patients are shown in Supplementary Table S6. Consistent with the above results, an increased p-NF-κB p65 level was associated with elevated NLRP3 expression, more infiltration of M1 macrophages (CD11B + CD86+), and less infiltration of M2 macrophages (CD11B + CD206+) (Fig. 7A–E). In this clinical cohort, we also found that the p-NF-κB p65 and NLRP3 protein levels and M1 macrophage infiltration were increased in the tumor tissues of patients who responded to immunotherapy, while they were reduced in those who did not respond to immunotherapy (Fig. 7F–M). An overview of APG-2575’s mechanisms is presented in Fig. 8.

## Discussion

BCL-2 inhibitors have transformed the treatment of hematological malignancies through direct induction of tumor cell apoptosis. In this study, we first found that the BCL-2 inhibitor APG-2575 enhanced the potent antitumor effects of ICIs on non-small cell lung cancer through its previously unreported ability to augment immune responses. This suggests that the combination of APG-2575 with anti-PD-1 immunotherapy may be a promising therapeutic strategy.

BCL-2 family members have been implicated in the proliferation and apoptosis of tumor cells and immune cells [21]. The effects of BCL-2 on immune cells were previously focused on T cells, in which it can increase longevity and promote the functions of effector T cells [39]. Because of its extensive effects on immune cells and regulation of programmed cell death, BCL-2 has become an attractive drug target for cancer therapy [40]. Frederick J Kohlhapp et al. observed that the BCL-2 inhibitor venetoclax decreased the proportion of naïve-like T cells but simultaneously elevated the proportion of CD8+ T effector memory cells to exert synergistic effects with ICIs [21]. However, the underlying mechanism by which BCL-2 inhibitors enhance the recruitment and cytotoxic functions of T cells remains unknown.

Macrophages play an essential role in the immune system and the cancer immunotherapy response. M1 macrophages have generally been described as proinflammatory macrophages, as they have antitumor characteristics. While the presence of a large proportion of TAMs can facilitate tumor immune escape and protumor effects, phenotypically, TAMs resemble M2 macrophages, which is why they have been called anti-inflammatory macrophages [41]. There are relatively few reports on the regulation of macrophages by BCL-2 inhibitors. Our study is the first to show that APG-2575 improves the efficacy of ICIs by polarizing M2 macrophages toward the M1 phenotype to promote CD8+ T-cell infiltration. In support of these encouraging preclinical results, additional studies have demonstrated that BCL-2 is essential for T-cell survival [42, 43] but not for memory T-cell survival. APG-2575 could also reduce the population of naive T cells and promote antitumor activity by increasing the population of effector T cells via a unique mechanism with immune regulation at its core. By regulating macrophage polarization, APG-2575 may have potential use as part of a clinical combination to improve the efficacy of PD-1 blockade.

Based on the results presented here, APG-2575 induced M1 macrophage polarization, which enhanced CD8+ T-cell infiltration into the TME via the chemokines CCL5 and CXCL10. This is consistent with a recent study showing that CD8 expression in lung adenocarcinoma is positively correlated with CCL5 and CXCL10 expression [44]. Among all known human chemokines, CCL4, CCL5, CXCL9 and CXCL10 are also strongly associated with CD8+ T-cell infiltration [45]. Although BCL-2 inhibitors have been widely studied for their groundbreaking effects in the treatment of hematological neoplasms [46], little is known about targeting BCL-2 for regulating chemokines during the antitumor immune response. Only one study has demonstrated that the CXCL12/CXCR4/CD44 axis can promote resistance to venetoclax-induced apoptosis in human AML cells [47]. Together, these findings support the idea that APG-2575 has an important effect on enhancing CCL5- and CXCL10-mediated CD8+ T-cell recruitment into the TME to promote tumor regression. Our results raise the possibility that the promotion of CCL5 and CXCL10 secretion may be the dominant mechanism by which APG-2575 exerts its antitumor effects.

Since APG-2575 is a BCL-2 inhibitor, our initial goal was to use APG-2575 to investigate the regulatory effects of BCL-2 on macrophages. However, we observed that BCL-2 upregulation or downregulation in macrophages or tumor cells did not impact the polarization of M2-like macrophages to the M1-like phenotype, indicating that APG-2575 induces macrophage polarization in a BCL-2-independent manner. Consistent with our findings, others have recently shown that venetoclax exhibits inhibitory activity toward the electron transport chain (ETC) independent of its intended target BCL-2, and this activity may contribute to the synergistic effect of venetoclax and ETC inhibitors in patients with hematological neoplasms [48–50]. Moreover, Cortecka et al. found that midostaurin, a PKC inhibitor, can exhibit a synergistic antitumor effect with inhibitors of its off-target PLK1 in NSCLC cells [51, 52]. Because tumors exhibit polygenicity and involve complex biological signaling networks and feedback loops, with small molecules often causing off-target effects [53–59], the regulation of macrophage polarization by APG-2575 is also an unexpected off-target effect. Utilizing such beneficial off-target effects could therefore result in new and promising precision medicine approaches [60].

To discover how APG-2575 regulates macrophage polarization, we conducted mechanistic experiments. They revealed that APG-2575 activated the NF-κB/NLRP3 signaling pathway to polarize M2 macrophages toward the M1 phenotype. NF-κB is involved in a variety of biological processes, from the immune response to cell proliferation and apoptosis, which have become the focus of disease and drug development [61, 62]. Gu et al. noted that the activation of the NF-κB pathway may play a positive regulatory role in the immune response by sensitizing tumor cells to T-cell-mediated killing [63]. NF-κB is also a key transcription factor in M1 macrophages and is required for the induction of numerous inflammatory genes [64]. The activation of NF-κB could also promote M1 polarization and indirectly enhance the adaptive immune response to induce regression of melanoma [65]. Strikingly, metformin can activate the AMPK-NF-κB signaling pathway and induce the polarization of M2 macrophages to M1 macrophages, thereby generating an antitumor effect [66]. PI3K inhibition has also been shown to activate the NF-κB signaling pathway and promote proinflammatory polarization of macrophages to suppress tumor growth [67]. Consistent with these previous investigations, we found that APG-2575-induced M1 macrophage polarization was significantly related to NF-κB signaling. We further confirmed that NF-κB plays an important role in the regulation of macrophage polarization. NF-κB is a central mediator of the initiation signal of NLRP3 inflammasome activation and acts by inducing the transcription of NLRP3 and pro-IL-1β in response to various cytokines [68]. We identified for the first time that the BCL-2 inhibitor APG-2575 induces NLRP3 upregulation and IL-1β production in M1 macrophages by activating the NF-κB pathway. NLRP3 has previously been identified as a double-edged sword in oncogenesis and influences the immune response by regulating host immunity. Melanoma patients with an NLRP3 mutation were found to exhibit increased infiltration of immune response cells, which was associated with better clinical efficacy [69]. In contrast, NLRP3 signaling in TAMs in pancreatic cancer drives macrophage-induced immunosuppression, thereby inhibiting CD8+ T-cell activation [70]. Consistent with previous data [65], we found that the activation of NF-κB/NLRP3 after APG-2575 treatment polarized M2 macrophages toward the M1 phenotype and enhanced their antitumor effects. Therefore, it is of great importance to understand how APG-2575 activates NF-κB/NLRP3 signaling to regulate macrophage polarization.

We next demonstrated that APG-2575 activated the NF-κB/NLRP3 signaling pathway through its interaction with NF-κB p65 in macrophages, again independent of its target BCL-2. Several studies have shown that NF-κB is central to the regulation of BCL-2 in renal cancer cells [71] and nude mouse lung xenografts [72]. However, the interactions of BCL-2 inhibitors with NF-κB p65 are underexplored. To our knowledge, our study is the first to show that APG-2575 can directly bind the NF-κB p65 protein to activate the NF-κB/NLRP3 signaling pathway. Recently, the effects of the binding of drugs and small molecules, namely, whether they are inhibitory or activating, has become a hot topic in cancer research [73, 74]. A recent molecular docking study clarified that Gremlin1 binds to fibroblast growth factor receptor 1 (FGFR1) and then activates the MAPK signaling pathway, an effect that synergizes with androgen deprivation therapy (ADT) in castration-resistant prostate cancer (CRPC) [73]. In contrast, venetoclax can interact with the NOS2 protein and has a good inhibitory binding effect on NOS2, making it a potential drug for reducing NOS2 expression in cervical cancer tumor cells [75]. However, no study has reported that BCL-2 inhibitors activate the NF-κB pathway through directly binding to NF-κB p65, and this result may provide a new potential mechanism for treatment with other BCL-2 inhibitors.

In summary, our results demonstrate that APG-2575 activates NLRP3 through interaction with NF-κB p65 to reset M2 macrophages to the M1 phenotype and promotes CD8+ T-cell infiltration through the chemokines CCL5 and CXCL10, further enhancing the efficacy of immunotherapy. For the first time, we showed that APG-2575 directly binds to NF-κB p65 to activate NLRP3 signaling, thereby inducing macrophage polarization. This may be a unique yet feasible mechanism supporting the treatment of cancer patients with BCL-2 inhibitors. Our study provides preclinical evidence for a novel potential combination strategy for evaluation in future clinical trials.

## Materials and methods

### Cell lines and reagents

The human NSCLC cell line (H1299) was purchased from the American Type Culture Collection (ATCC, USA), the human monocytic cell line THP-1 and the murine macrophage line RAW264.7 were obtained from the laboratory of Dr. Dajun Yang, and the murine Lewis lung carcinoma (LLC) cell line and the HEK293 cell line were obtained from the laboratory of Dr. Liwu Fu. All cell lines were cultured with RMPI-1640 medium (Life Technologies) or high-glucose Dulbecco’s modified Eagle’s medium (Corning) supplemented with 10% fetal bovine serum (FBS) (Gibco, Australia) at 37 °C in 5% CO_2_. Cells were tested for mycoplasma and interspecies cross-contamination and were authenticated by isoenzyme and short tandem repeat (STR) analyses at Shanghai Biowing Applied Biotechnology Co., Ltd. (Shanghai, China) before the study and randomly during the research. APG-2575 was provided by Ascentage Pharma Group Inc. (Jiangsu, China). PMA and LPS were purchased from Sigma‒Aldrich. Mouse interleukin 4 (IL-4), mouse interferon gamma (IFN-γ), recombinant mouse macrophage colony-stimulating factor (M-CSF), mouse granulocyte-macrophage colony-stimulating factor (GM-CSF), human IL-4, human IFN-γ, human M-CSF, and human GM-CSF were purchased from PeproTech (Rocky Hill, NJ, USA). JSH-23, INF39 and the human drug pembrolizumab were purchased from Selleck Chemicals (Houston, TX, USA). For the in vivo experiments, a mouse anti-PD-1 antibody (clone CD279), anti-CD3 antibody (1452-C11), anti-CD4 antibody (GK1.5), anti-CD8 antibody (53-6.7) and isotype control (IgG) were purchased from Bio X Cell (West Lebanon, NH). The mouse anti-CCL5 neutralizing antibody was purchased from R&D Systems. The mouse anti-CXCL10 (IP-10) neutralizing antibody was purchased from PeproTech (Princeton, NJ, USA). The human anti-CCL5 and anti-CXCL10 antibodies were purchased from R&D Systems. The macrophage depletion reagent PLX3397 was purchased from Medkoo Biosciences (Chapel Hill, Cary, NC). The reagents used in this study are listed in Supplementary Table S1.

### Animal experiments

All animal protocols were performed following the National Institutes of Health (NIH) Guidelines for the Care and Use of Laboratory Animals and were approved by the institutional animal care and use committee of Sun Yat-Sen University Cancer Center. NSG mice with reconstitution of hematopoietic stem cells (HSCs) were purchased from Shanghai Model Organisms Center, Inc. (China) and housed under pathogen-free conditions. Female C57BL/6 and BALB/c nude mice (6–8 weeks old) were purchased from Vital River Laboratory Animal Technology Co., Ltd. (Beijing, China) and reared in a specific pathogen-free (SPF) barrier facility at the Animal Center of Sun Yat-sen University Cancer Center.

LLC and H1299 tumor cells were subcutaneously injected to establish tumor models in mice. The mice were treated with drugs when the tumor volume reached approximately 100 mm^3^. To generate subcutaneous H1299 non-small cell lung tumors in humanized mice, 5 × 10^6^ H1299 cells were subcutaneously implanted into the right flanks of female humanized NSG mice. The mice were randomly divided into four groups and treated with phosphate-buffered saline (PBS), APG-2575, an anti-PD-1 antibody, or APG-2575+the anti-PD-1 antibody. In the LLC subcutaneous C57BL/6 model, 5 × 10^5^ LLC cells were subcutaneously implanted into the right flanks of female C57BL/6 mice. One week later, the mice were randomly divided into groups and treated with PBS, APG-2575, an anti-PD-1 antibody, APG-2575+the anti-PD-1 antibody, an anti-CD3 neutralizing antibody, an anti-CD4 neutralizing antibody, an anti-CD8 neutralizing antibody, an anti-CCL5 neutralizing antibody, an anti-CXCL10 neutralizing antibody, isotype control, PLX3397, JSH-23, or INF39 at the indicated times. To generate subcutaneous H1299 or LLC tumors in BALB/c nude mice, 5 × 10^6^ H1299 cells or 5 × 10^5^ LLC cells, respectively, were subcutaneously implanted into the right flanks of female BALB/c nude mice. The mice were randomly divided into two groups and treated with PBS or APG-2575.

For the in vivo experiments, APG-2575 was formulated in 10% ethanol + 30% PEG 400 + 60% Phosal® 50PG. The human drug pembrolizumab, the mouse anti-PD-1 antibody (clone CD279) and mouse IgG1 (clone MOPC-21; BioXcell, Lebanon, NH, USA) were formulated in 1 × PBS. The drug administration strategy is described in Supplementary Table S2. Subcutaneous tumor growth was monitored by measuring the length (*L*) and width (*W*) of the tumors using Vernier calipers. The tumor volume (V) was calculated using the equation *V* = (*L* × *W*^2^)/2. Tumor growth in the subcutaneous tumor model was monitored every two days, and the survival of tumor-bearing mice was evaluated every day. When the experimental endpoints were met or the tumor volume reached 2000 mm^3^, all of the mice were euthanized according to NIH guidelines. The tumors were resected and stored in MACS Tissue Storage Solution on ice (Miltenyi Biotec, Auburn, CA, USA). The tumors from all experiments were then processed on the same day for FACS analysis or sorting. The remaining collected tumors and organs were fixed in 10% paraformaldehyde, embedded in paraffin, sliced into ~4 μm sections, and stained with hematoxylin and eosin (H&E).

### Preparation of single-cell suspensions from tumors

The isolation of tumor-infiltrating cells has previously been described [76], but the procedure was slightly modified for this study. Briefly, tumor tissues were collected and cut into small pieces in PBS. After centrifugation, enzymatic digestion was performed using a prepared enzyme mix from a tumor dissociation kit (Miltenyi Biotec, Auburn, CA, USA) with a gentle MACS dissociator (Miltenyi Biotec) for 1 h at 37 °C. Next, the cell suspensions were filtered through a 70-μm cell strainer (Becton Dickinson). Red blood cells were lysed with ACK lysis buffer prior to washing with FACS buffer. The cell suspensions were then subjected to centrifugation with Ficoll to harvest the mononuclear cells and/or sorted with anti-F4/80 microbeads (Miltenyi Biotec) to harvest the tumor-infiltrating macrophages.

### Flow cytometric analysis

For surface marker analysis, live cells were resuspended in 1 × PBS and stained with antibodies at 4 °C for 30 min. The concentration of each antibody used was determined according to the recommended product protocol. In some cases, cells were pretreated with a mouse anti-CD16/32 antibody (BioLegend, #101320) to block nonspecific binding of immunoglobulin to macrophage Fc receptors. For intracellular cytokine staining, cells were fixed and permeabilized after stimulation with Cell Activation Cocktail (with Brefeldin A) (Biolegend, #423303) in an incubator for 6 h and labeled with antibodies. The cells were then fixed and permeabilized without stimulation. Data were analyzed with FlowJo software. The Abs used for flow cytometric analyses were purchased from eBioscience, BioLegend, and BD Biosciences (Supplementary Table S1).

### Generation of mouse bone marrow-derived macrophages (BMDMs) and human macrophages

As described elsewhere [77], bone marrow cells were isolated from the femurs of female C57BL/6 mice and cultured with 20 ng/ml recombinant M-CSF (PeproTech) for 5 days. On Day 6, naive BMDMs were collected and then stimulated for 24 h with 20 ng/ml IL-4 (PeproTech) or 100 ng/ml LPS (Sigma‒Aldrich) plus 20 ng/ml IFN-γ (PeproTech) to generate BMDM-M2s or BMDM-M1s, respectively. For human macrophage culture, monocytes were isolated from the blood of healthy donors by magnetic bead separation (Miltenyi Biotec) and cultured with 20 ng/ml recombinant human M-CSF (PeproTech) to induce differentiation into macrophages. Seven days later, 20 ng/ml recombinant human IL-4 (PeproTech) was added to induce M2 polarization of these macrophages.

### T-cell proliferation and tumor cytotoxicity assays

T cells were isolated from the spleens of female C57BL/6 mice using a Pan T-Cell Isolation Kit (Miltenyi Biotec, CA, USA). Then, carboxyfluorescein succinimidyl ester (CFSE) (Sigma‒Aldrich, MO, USA)-labeled T cells were cultured with 10 ng/ml IL-2 (PeproTech, NJ, USA) in complete RPMI-1640 medium (10% FBS, 100 U/ml penicillin–streptomycin) and stimulated with CD3/CD28 T-Cell Activator (Stemcell Technologies, BC, Canada) in the presence or absence of conditioned medium from control or APG-2575-treated BMDM-M2 cells. After 72 h, CFSE was detected, and T cells were labeled with CD8 for specific measurement of T-cell proliferation. In some instances, macrophage-induced tumor cell apoptosis or APG-2575-induced macrophage apoptosis was measured by Annexin-V and PI staining (BD Pharmingen).

### Reverse transcription qPCR

For quantitative reverse transcription polymerase chain reaction (qRT‒PCR) analysis, cells were harvested and processed for RNA extraction. Total RNA (1 μg) was extracted from cells or tumor tissues with TRIzol reagent (Invitrogen, USA) and reverse transcribed into cDNA using the ReverTra Ace Kit (Yishan Biotechnology Co., Ltd., Shanghai, China). cDNA was amplified using SYBR Green qPCR Master Mix (ROX2 plus) (EZBioscience, Roseville, USA) on a Real-Time PCR System (Roche Applied Science, Penzberg, Germany). The target mRNA levels were normalized to those of GAPDH or β-actin. The primer sequences are listed in Supplementary Table S3.

### ELISA

The concentration of secreted IL-10 in culture medium from IL-4-activated CD14+ monocyte-derived macrophages and mouse BMDMs was measured using human and mouse ELISA kits (RayBiotech) according to the manufacturer’s protocol.

### CBA assay

Quantitative determination of the TNF-α, CCL5, and CXCL10 concentrations in samples was performed using the CBA Human Cytokine Kit and CBA Mouse Cytokine Kit (BD Biosciences; San Diego, CA, USA) according to the manufacturer’s protocol.

### Western blot analysis

Total cellular protein in cell lysates and proteins in the prestained Protein Ladder (Thermo Fisher Scientific) were separated by 10% SDS-polyacrylamide gel electrophoresis and then transferred onto nitrocellulose membranes. After blocking with 5% bovine serum albumin (BSA) in Tris-buffered saline containing 0.1% Tween 20 for 1 h at room temperature, the membranes were incubated with different primary antibodies. For details, see Supplementary Table S1. Next, the membranes were washed and incubated with horseradish peroxidase-conjugated secondary antibodies. The Nuclear/Cytosol Fractionation Kit (Thermo Fisher, USA) was used according to the manufacturer’s protocol. In some instances, during the extraction of cytoplasmic and nuclear proteins, the proteins were visualized using electrochemiluminescence (ECL) western blotting reagent (Thermo Pierce).

### Immunofluorescence and immunohistochemical staining

For immunofluorescence staining, cells were seeded in a confocal dish for 24 h with or without APG-2575 treatment. In some cases, other compounds were added for pretreatment. Cells were fixed with 4% paraformaldehyde in PBS (pH 7.4) for 10 min at room temperature and then permeabilized with 0.5% Triton X-100 in PBS for 10 min. After blocking with 2% BSA in PBS containing 0.1% Tween 20 for 30 min, the cells were incubated with antibodies specific for NF-κB p65 (Cell Signaling Technology, #8242, 1: 100), NOD-like receptor family pyrin domain containing 3 (NLRP3) (Bioss Inc., Beijing, #bs-10021R, 1:100), and adaptor apoptosis-associated speck-like protein (ASC) (Bioss Inc., Beijing, bs-6741R, 1:100) in PBS containing 2% BSA and 0.1% Tween 20 overnight at 4 °C. After the cells were washed and stained with a secondary antibody for 1 h at room temperature, nuclei were stained with DAPI (4’,6-diamidino-2-phenylindole) (2 μg/ml). A fluorescence microscope (Olympus BX51, Tokyo, Japan) was used to observe the slides and acquire merged images.

For immunohistochemical staining, lung cancer tissues were isolated from tumor-bearing mice, fixed in 37% formalin and embedded in paraffin. The sections were then incubated with various primary antibodies, which are detailed in Supplementary Table S1. Subsequently, the sections were incubated with an anti-rabbit secondary antibody (ZsBio, China) after being washed with PBS, and staining was then visualized with DAB (ZsBio, China).

### Chemotaxis assay

The chemotaxis of murine and human CD8+ T cells was assayed in 24-well plates (5-μm-pore-size Transwell inserts with polycarbonate membranes; Corning). Medium alone (RPMI 1640 plus 10% FBS) or macrophage culture supernatants were added to the bottom compartments of triplicate wells. Murine or human T-cell migration was assessed with medium, APG-2575-treated supernatant alone, supernatant plus 10 μg/mL anti-CCL5 neutralizing antibodies, and/or 10 μg/mL anti-CXCL10 neutralizing antibody (R&D Systems). CD8+ T cells (5 × 10^5^) from C57BL/6 mouse spleens or the blood of healthy donors were purified with mouse or human anti-CD8a beads (Miltenyi Biotec), placed in Transwell inserts and incubated at 37 °C for 8–12 h. Cells in the bottom compartments were enumerated by flow cytometry. The numbers of spontaneously migrated cells were subtracted from the total number of migrated cells under all conditions, and the data are reported as the chemotactic index. Chemotactic index = (migrated cells – spontaneously migrated cells)/total T cells plated in the Transwell insert × 100%.

### Electrophoretic Mobility Shift Assay (EMSA)

For the EMSA, we obtained the nuclear and cytoplasmic protein fractions using a cytoplasmic/nuclear protein extraction kit (Thermo Fisher, USA). The DNA–protein complexes were loaded onto 4% nondenaturing polyacrylamide gels. After 35 min of separation by electrophoresis in Tris borate-EDTA buffer, the products in the gels were transferred onto Hybond-N+ membranes. The signals were detected using the Bio-Rad ChemiDoc XRS+ system. The probe sequences are presented in Supplementary Table S4.

### Luciferase reporter assay

Cells were transfected with 500 ng of luciferase reporter plasmids and 50 ng of the pRL-TK vector (an internal control with a Renilla luciferase gene). Transfection was performed using Lipo3000 according to the manufacturer’s protocol. After 24 h, luciferase activity was measured with a Dual Luciferase Reporter Assay System (Promega) according to the manufacturer’s instructions. The relative luciferase activity values in the treated cells were normalized to those in the control cells.

### Chromatin immunoprecipitation (ChIP) assay

ChIP assays were performed as previously described [78]. ChIP assays were conducted using an Enzymatic Chromatin IP Kit (Magnetic Beads). Cells were cultured in 10-cm dishes and treated with APG-2575 (10 μM), the combination of APG-2575 and JSH-23, or DMSO. Cells were then subjected to crosslinking with 1% formaldehyde for 20 min at room temperature. After termination of crosslinking by the addition of glycine, DNA was digested with micrococcal nuclease, and chromatin was sheared and was then immunoprecipitated with an anti-NF-κB p65 antibody or normal rabbit IgG. Immunoprecipitated chromatin was decrosslinked at 65 °C for 4 h and purified using spin columns. Primer sequences:

NLRP3: Forward: 5′-GAGCCCTGAGGTTTCACTTTTTCCCATTG-3′

NLRP3: Reverse: 3′- GGTTAGGCAGAAACTGTCACTACGTTCGA-5′.

### Flow cytometric analysis of apoptotic cells

LLC cells, H1299 cells, BMDM-M1s (induced by LPS + IFN-γ), BMDM-M2s (induced by IL-4) and BMDM-TAMs (induced by conditioned medium (CM) from tumor cells) were seeded at a density of 1 × 10^6^ cells/well in 6-well plates. After the cells were cultured for 24 h, APG-2575 was added to the culture plates, and the cells were then cultured under routine conditions. Twenty-four hours later, the cells were collected and washed with PBS. Annexin V/FITC and PI were added to the cells for staining 15 min. Subsequently, the stained cells were analyzed by flow cytometry (BD Biosciences, San Jose, CA, USA).

### Biotinylated protein interaction pulldown assay

Biotinylated APG-2575 (Bio-APG-2575) and biotin were purchased from Wayen Biotech (Shanghai, China). For the pulldown assay, we used PierceTM Biotinylated Protein Interaction Pull-Down Kits (Thermo Fisher). Lysates prepared from BMDMs and RAW264.7 cells were added to streptavidin-agarose beads with bio-APG-2575. Lysates prepared from BMDMs and RAW264.7 cells transfected with the wild-type NF-κB p65 or mutant NF-κB p65 construct were also added. The prepared samples were loaded onto a polyacrylamide gel for protein separation and subsequent Western blotting.

### Evaluation of APG-2575 binding sites on the NF-κB p65 protein

The plasmids pcDNA3.1-Flag-NF-κB p65 (mouse, WT) and pcDNA3.1-Flag-NF-κB p65 (mouse, Arg33A/Lys56A/Asp277A/Arg278A) were provided by Saisofi Biotechnology Co., Ltd. (Suzhou, China). BMDMs and RAW264.7 cells were transfected with these plasmids, and lysates were prepared for use in biotinylated protein interaction pulldown assays.

### Macrophage migration assay

Macrophage migration assays were conducted by using 24-well Transwell inserts (8 μm; BD Biosciences). Briefly, IL-4-activated BMDMs and RAW264.7 cells were starved overnight. Then, a suspension of 4 × 10^4^ cells was placed into the upper compartments. The macrophages were pretreated with or without APG-2575 for 24 h. After the macrophages were allowed to migrate for 24–36 h, methanol fixation (10 min) and crystal violet staining (15 min) were performed. Cells were enumerated using ImageJ (NIH, Bethesda, MD, USA).

### Patients and non-small cell lung cancer samples

Primary human NSCLC samples were obtained from 53 patients with advanced NSCLC who received immunotherapy during their course of anticancer therapy at Sun Yat-sen University Cancer Center (Guangzhou, China). This study was performed with permission from the Ethics Committee of Sun Yat-sen University Cancer Center’s Institutional Review Board.

### Tissue multiplex immunohistochemistry

NSCLC samples were stained with a multiplex fluorescence immunohistochemical kit, PDOne six-color TSA-RM-82758 (100 T) (cat 10234100100 Panovue, Beijing, China). After incubation with the primary antibody, the samples were incubated with an HRP-conjugated secondary antibody, and tyrosine signal amplification (TSA) was performed to label antigens. After each TSA labeling step, the primary and secondary antibodies were removed using a microwave for heat-induced antigen retrieval. After the sample was eluted, the next antigen was labeled, and this procedure was repeated for all five antigen markers. Nuclei were stained with 4′-6′-diamidino-2-phenylindole (DAPI, Sigma‒Aldrich) when immunohistochemical staining was complete. The antibodies used for staining were anti-NF-kB p65 (polyclone, dilution 1:600, Signalway Antibody), anti-NLRP3 (polyclone, dilution 1:800, Proteintech), anti-CD86 (clone E2G8P, dilution 1:1000, Cell Signaling Technology), anti-CD206 (clone E6T5J, dilution 1:200, Cell Signaling Technology), and anti-PANCK (clone C11, dilution 1:200, Cell Signaling Technology). Lung cancer samples were scanned, and fluorescence images were acquired at ×20 magnification with a PanoVIEW VS200 slide scanner (Panovue, Beijing, China) and an Olympus 20× lens. Image recognition and analysis were performed with QuPath image analysis software (Version 0.3.0, Queen’s University of Belfast, Northern Ireland, UK). The images were quantized into data by tissue segmentation and cell segmentation using the positive threshold settings and phenotypic recognition. The quantitative data were assembled by an R script (Version 4.1.2), and basic data such as the positive cell number, positive staining rate and density were obtained for analysis.

### RNA-seq and gene enrichment analyses

Gene expression analysis was performed by RNA-seq for the conditions shown in the relevant figures. Cells subjected to different treatments were harvested for RNA extraction using TRIzol. The sequencing library was established after high-quality RNA was quantified and was subsequently sequenced on an Illumina NovaSeq platform. To demonstrate the differential gene expression between the control and treated samples, the expression level of each transcript was determined based on the fragments per kilobase of exon per million mapped reads method. Differential expression analysis was conducted using the R statistical package software EdgeR (http://www.bioconductor.org/packages/2.12/bioc/html/edgeR.html). Functional enrichment analyses involving Gene Ontology (GO) terms and KEGG pathways, as well as gene set enrichment analysis (GSEA), were also performed.

### Bioinformatic analysis of a public dataset and statistical analysis

Immune cell infiltration scores were systematically evaluated using the CIBERSORT [79] method based on a TCGA-LUAD cohort and RNA-seq data downloaded from the Tumor IMmune Estimation Resource 2.0 (TIMER 2.0 [80], http://timer.comp-genomics.org/) database. The clinical data were collected from the University of California Santa Cruz (UCSC) [81]. Xena browser (https://xenabrowser.net). Patients without survival information or immune cell infiltration scores were removed from further analyses. Correlation analysis of gene expression and immune infiltration was performed with TIMER 2.0.

Differences between continuous variables were analyzed with the Wilcoxon test. Correlation coefficients were calculated by Spearman correlation and distance correlation analyses. For corresponding survival analysis of patients based on immune cell infiltration, survival curves were generated using the Kaplan–Meier method. The log-rank (Mantel‒Cox) test was used to determine the statistical significance of differences. All P values were two-tailed, and a *P* value of < 0.05 was considered to indicate a statistically significant difference. All data analysis and image presentation tasks were carried out using R (version 4.1.0) and R Bioconductor packages.

### Molecular docking

Molecular docking was conducted in MOE v2018.01011. The 3D structure of the mouse RELA protein was downloaded from the RCSB PDB Data Bank (PDB ID 1IKN) [82]. The 2D structures of small molecules were converted to 3D structures in MOE through energy minimization. The binding sites of the native ligands in the protein structures were set as binding pockets for small molecules [83]. The protonation state of the target and the orientation of the hydrogen atoms were optimized using the QuickPrep module at a pH of 7 and a temperature of 300 K. Prior to docking, the AMBER10:EHT force field and the reaction field (R-field) implicit solvation model were selected. The position of the native ligand in the X-ray structure of each receptor was defined as the binding site. The docking workflow followed the “induced fit” protocol, in which the side chains of the receptor pocket were allowed to move according to the ligand conformations with a constraint on their positions. The weight used for tethering side chain atoms to their original positions was 10. All docked poses of molecules were ranked by London dG scoring, and force field refinement was then carried out on the top 30 poses, followed by rescoring with the GBVI/WSA dG scoring function. The top-ranked pose was selected as the final binding mode. The binding mode was visualized using PyMOL (www.pymol.org).

### Molecular dynamics (MD) simulation

MD simulation was performed in GROMACS (version 2020.6), with 1 simulation for each of the 20 complexes of APG-2575 with the mouse RELA protein. For the AMBER14SB2 force field and general AMBER force field (GAFF), 3 parameters were used for the protein and APG-2575 molecule. The partial atomic charges of APG-2575 were calculated with the restrained electrostatic potential (RESP)4 charges following the optimization of the molecule at the B3LYP/6-31 G(d) level using the Gaussian165 package. The complex was then neutralized by adding chlorine counterions and solvated in a box of TIP3P6 water molecules with solvent layers located at a distance of 1.2 nm between the box edges and solute surface. The particle mesh Ewald (PME)7 method was used to treat the long-range electrostatic interactions. The calculated radius of van der Waals interactions was 1.2 nm.

Before the production run, the systems were relaxed with 1000 steps using the steepest descent algorithm followed by another 1000 steps using the conjugate gradient method. For the equilibration phase, the temperature and pressure were controlled using the Berendsen coupling algorithm with time constants of 0.1 and 1.0 ps, respectively. For the production run, an integration time step of 2 fs was employed to integrate the equations of motion. The Parrinello-Rahman coupling algorithm was used to keep the pressure constant. The simulated temperature was set to 298.15 K, and 100 ns MD simulation was performed in the NPT ensemble. The binding free energy of each receptor–ligand complex was calculated with gmx_MMPBSA (version 1.4.3)10 based on MMPBSA.py11 in the AmberTools20 suite.

The molecular visualizations of the receptor–ligand complexes were created with VMD v1.9.4 (Visual Molecular Dynamics) software12. 2D diagrams of the receptor–ligand complexes were created with PoseView13.

### Single-cell RNA sequencing (scRNA-seq)

#### Sample collection

H1299 tumor cells (5 × 10^6^) were transplanted into humanized CD34+ model mice. Two weeks later, the tumors were harvested, and 0.1 g of tumor tissue from each tumor was collected. Single-cell suspensions were generated following the methods described below in the “Preparation of single-cell suspensions from tumors” section. A total of 5 × 10^3^ viable human CD45+ immune cells were sorted by flow cytometry.

#### scRNA-seq

Cell capture and cDNA synthesis were carried out using a single-cell 5′ Library and Gel Bead Kit (10x Genomics, 1000006) and a Chromium Single Cell A ChIP Kit (10x Genomics, 120236). The cell suspension (300-600 viable cells per microliter, as measured with a Countstar system) was loaded onto the Chromium single-cell controller (10x Genomics) to generate single-cell gel beads in emulsion according to the manufacturer’s protocol. In brief, single cells were suspended in PBS containing 0.04% BSA. Captured cells were lysed, and the released RNA was barcoded through reverse transcription in individual gel beads in emulsion (GEMs). Reverse transcription was performed on an S1000TM Touch Thermal Cycler (Bio-Rad) with the following thermal cycling program: 53 °C for 45 min, followed by 85 °C for 5 min and holding at 4 °C. cDNA was generated and then amplified, and the quality was assessed using an Agilent 4200 system (performed by CapitalBio Technology, Beijing).

#### Single-cell RNA-seq library preparation

ScRNA-seq library preparation was conducted according to the manufacturer’s instructions. ScRNA-seq libraries were constructed using a Single Cell 5’ Library and Gel Bead Kit, a Single Cell V(D)J Enrichment Kit, Human T Cells (1000005) and a Single Cell V(D)J Enrichment Kit, Human B Cells (1000016). The libraries were finally sequenced using the Illumina NovaSeq 6000 platform with a sequencing depth of at least 100,000 reads per cell with a paired-end 150 bp (PE150) strategy (performed by CapitalBio Technology, Beijing).

#### Data preprocessing

##### Cell Ranger pipeline

Cell Ranger software was obtained from the 10x Genomics website https://support.10xgenomics.com/single-cell-gene-expression/software/downloads/latest. Alignment, filtering, barcode counting, and UMI counting were performed with the Cell Ranger count module to generate a feature-barcode matrix and to identify clusters. Dimensionality reduction was performed using PCA, and the first ten principal components were used to generate clusters via the K-means algorithm and a graph-based algorithm.

##### Seurat pipeline

The other clustering pipeline used was Seurat 3.0 (R package). Cells containing fewer than 200 genes, ranked in the top 1%, or containing more than 25% mitochondrial DNA were regarded as abnormal and filtered out. Dimensionality reduction was performed using PCA, and data were visualized on TSNE and UMAP plots.

##### Cell type annotation

Clusters were annotated using manual analysis of cell type-specific gene markers obtained from the literature (Supplementary Table S5) in combination with automatic cell type identification using CellAssign [84] (http://bio-bigdata.hrbmu.edu.cn/CellMarker/). Cell type recognition from single-cell RNA sequencing data was performed by leveraging reference transcriptomic datasets of pure cell types to independently infer the cell of origin of each single cell. For annotation of human cell types, Blueprint_Encode or HPCA was used.

### Statistical analyses

All statistical analyses were performed using SPSS 22.0 (IBM, Armonk, USA) or GraphPad Prism (version 8.0). All experiments were carried out at least three times, and the data from one representative experiment are shown. The data are shown as the means ± SDs. The statistical tests used for data analysis were two-tailed Student’s t test, one- and two-way ANOVA, Fisher’s exact test, the chi-square test and the log-rank test. Differences with *P* values < 0.05 were regarded as statistically significant. *P < 0.05, **P < 0.01, ***P < 0.001, ****P < 0.0001. NS indicates a nonsignificant difference.

### Supplementary information

Supplementary materials

## Data Availability

The data generated and analyzed will be made from the corresponding author on reasonable request. The authenticity of this article has been validated by uploading the key raw data onto the Research Data Deposit public platform (www.researchdata.org.cn), with the approval RDD number as RDDB2023416127.
