# Supplementary material for: The BCL-2 inhibitor APG-2575 resets tumor-associated macrophages toward the M1 phenotype, promoting a favorable response to anti-PD-1 therapy via NLRP3 activation
Source: Cell Mol Immunol. 2023 Dec 7;21(1):60–79. doi: 10.1038/s41423-023-01112-y (PMC10757718; doi:10.1038/s41423-023-01112-y)
Supplement: Supplementary file 1 — Supplementary materials [file 41423_2023_1112_MOESM1_ESM.docx]

**Supplementary Table S1. Reagents used in this study.**

| **REAGENT or RESOURCE** | **IDENTIFIER** | **SOURCE** |
| --- | --- | --- |
| APG-2575 | - | Ascentage Pharma |
| Phorbol 12-myristate 13-acetate | Cat# P1585 | Sigma-Aldrich |
| Lipopolysaccharide | Cat# SMB00610 | Sigma-Aldrich |
| JSH-23 | Cat# S7351 | Selleck Chemicals |
| INF39 | Cat# S8559 | Selleck Chemicals |
| PLX3397 | Cat# 206178 | Medkoo Biosciences |
| Human M-CSF | Cat# BGK09603 | PeproTech |
| Human GM-CSF | Cat# 300-03 | PeproTech |
| Human IL-4 | Cat# 200-04 | PeproTech |
| Human IFN-γ | Cat# AF-300-02 | PeproTech |
| Human pembrolizumab | Cat# A2005 | Selleck Chemicals |
| Human CCL5 Antibody | Cat# MAB278-100 | R&D Systems |
| Recombinant Murine IL-4 | Cat# 214-14 | PeproTech |
| Recombinant Murine IFN-γ | Cat# 315-05 | PeproTech |
| Recombinant Murine M-CSF | Cat# 315-02 | PeproTech |
| Recombinant Murine GM-CSF | Cat# 315-03 | PeproTech |
| Mouse CCL5 Antibody | Cat# MAB478 | R&D Systems |
| Mouse CXCL10 Antibody | Cat# MAB466 | R&D Systems |
| Anti-mouse PD-1 (CD279) | Cat# BP0146 | Bio X Cell |
| Anti-mouse CD3 (145-2C11) | Cat# BE0001 | Bio X Cell |
| InVivoMAb mouse IgG2a isotype control (C1.18.4) | Cat# BE0085 | Bio X Cell |
| Anti-mouse CD4 (GK1.5) | Cat# BP0003 | Bio X Cell |
| InVivoPlus mouse IgG2b isotype control (LTF-2) | Cat# BP0090 | Bio X Cell |
| Anti-mouse CD8 (53-6.7) | Cat# BP0004 | Bio X Cell |
| InVivoPlus mouse IgG2a isotype control (2A3) | Cat# BP0089 | Bio X Cell |
| GentleMACS 25 C Tubes | Cat# 130-093-237 | Miltenyi Biotec |
| Enzyme digestion | Cat# 130-096-730 | Miltenyi Biotec |
| Anti-F4/80 Microbeads Ultrapure, mouse | Cat# 130-110-443 | Miltenyi Biotec |
| Pan T Cell Isolation Kit, human | Cat# 130-096-535 | Miltenyi Biotec |
| CFSE | Cat# 92846 | Sigma-Aldrich |
| Recombinant Human IL-2 | Cat# 200-02 | PeproTech |
| CD3/CD28 T Cell Activator | Cat# 10970 | Stemcell Technologies |
| MACS® Comp Bead Kits | Cat# 130-104-693 | Miltenyi Biotec |
| FITC Annexin V Apoptosis Detection Kit I | Cat# 556547 | BD Pharmingen |
| Tumor Dissociation Kit, mouse | Cat# 130-096-730 | Miltenyi Biotec |
| Lipofectamine® 3000 Reagent | Cat# L3000015 | Invitrogen |
| ***Continued*** |  |  |
| **REAGENT or RESOURCE** | **IDENTIFIER** | **SOURCE** |
| Opti-MEM® Medium | Cat# 11058021 | Gibco |
| ABT-199 | Cat# S8048 | Selleck Chemicals |
| BDA-366 | Cat# S7849 | Selleck Chemicals |
| **Reagents used in qPCR** | | |
| TRIzol™ Reagent | Cat# 15596018 | Invitrogen |
| Fast All-in-One RT Kit (with gDNA Remover) | Cat# ES-RT001 | Yishan Biotechnology |
| Color SYBR Green qPCR Mix (ROX2 plus) | Cat# A0012 | EZBioscience |
| **Reagents used in ELISA** | | |
| Mouse IL-10 ELISA Kit | Cat# ELM-IL10-1 | RayBiotech |
| Human IL-10 ELISA Kit | Cat# ELH-IL10-1 | RayBiotech |
| **Reagents used in western blot** | | |
| NF-κB p65 H&M | Cat# 8242 | Cell Signaling Technologies |
| IL-1β H&M | Cat# 12242 | Cell Signaling Technologies |
| Arginase-1 H&M | Cat# 93668 | Cell Signaling Technologies |
| iNOS H&M | Cat# bs-0162R | Bioss Inc. |
| NLRP3 H&M | Cat# bs-10021R | Bioss Inc. |
| ASC H&M | Cat# bs-6741R | Bioss Inc. |
| Histone H3 H&M | Cat# 4499 | Cell Signaling Technologies |
| GAPDH H&M | Cat# 5174 | Cell Signaling Technologies |
| Caspase-1 H | Cat# 3866 | Cell Signaling Technologies |
| Caspase-1 M | Cat# 24232 | Cell Signaling Technologies |
| NE-PER Extraction reagents | Cat# 78833 | Thermo Fisher |
| Phospho-NF-κB p65 H&M | Cat# 3033 | Cell Signaling Technologies |
| TRIF H&M | Cat# abs159876 | Absin |
| MyD88 H&M | Cat# ab219413 | Abcam |
| TRAF6 H&M | Cat# ab33915 | Abcam |
| SAPK/JNK H&M | Cat# 9252 | Cell Signaling Technologies |
| Phospho-SAPK/JNK H&M | Cat# 9251 | Cell Signaling Technologies |
| ERK H&M | Cat# 4695 | Cell Signaling Technologies |
| Phospho-ERK H&M | Cat# 9101 | Cell Signaling Technologies |
| P38 H&M | Cat# 8690 | Cell Signaling Technologies |
| Phospho-P38 H&M | Cat# 4511 | Cell Signaling Technologies |
| NLRC4 H&M  H&M | Cat# abs117861 | Absin |
| NLRP7 H&M | Cat# LS-C747040 | LSBio |
| BCL-2 H&M | Cat# ab182858 | Abcam |
| Flag H&M | Cat# 14793 | Cell Signaling Technologies |
| β-Actin H&M | Cat# 4970 | Cell Signaling Technologies |
| ***Continued*** |  |  |
| **REAGENT or RESOURCE** | **IDENTIFIER** | **SOURCE** |
| **Reagents used in IHC** |  |  |
| NLRP3 H&M | Cat# bs-10021R | Bioss Inc. |
| ASC H&M | Cat# bs-6741R | Bioss Inc. |
| CD8 H&M | Cat# ab237709 | Abcam |
| Granzyme B H&M | Cat# ab4059 | Abcam |
| CD86 H&M | Cat# 32223 | Signalway Antibody |
| CD206 H&M | Cat# ab64693 | Abcam |
| NF-κB p65 H | Cat# 11217 | Signalway Antibody |
| CD8 H | Cat# ab237710 | Abcam |
| Granzyme B H | Cat# BM5063 | BOSTER |
| CD86 H | Cat# ab270719 | Abcam |
| CD206 H | Cat# 91992S | Cell Signaling Technologies |
| NF-κB p65 H&M | Cat# 8242 | Cell Signaling Technologies |
| CD4 M | Cat# 25229 | Cell Signaling Technologies |
| CD4 H | Cat# 48274 | Cell Signaling Technologies |
| **Antibodies used in Flow** **cytometry** | | |
| Brilliant Stain Buffer | Cat# 563794 | BD Pharmingen |
| BV421 Hamster Anti-Mouse | Cat# 748259 | BD Pharmingen |
| CD11b APC-R700 M1/70 | Cat# 564985 | BD Pharmingen |
| Cytofix/Cytoperm W/GolgiPlug Kit | Cat# 555028 | BD Pharmingen |
| Rat/Ham Ig Kpa Comp Bead Set | Cat# 552845 | BD Pharmingen |
| Fixable Viability Stain 510 | Cat# 564406 | BD Pharmingen |
| Hu CD68 BV785 | Cat# 333826 | Biolegend |
| Hu CD11b/MAC-1 BV605 ICRF44 | Cat# 562721 | BD Pharmingen |
| Hu CD206 BV421 19.2 | Cat# 564062 | BD Pharmingen |
| Hu CD3 FITC UCHT1 | Cat# 555332 | BD Pharmingen |
| Hu CD4 PerCP-Cy5.5 RPA-T4 | Cat# 560650 | BD Pharmingen |
| Hu CD45 APC-H7 2D1 | Cat# 560178 | BD Pharmingen |
| Hu CD8 BV650 RPA-T8 | Cat# 563821 | BD Pharmingen |
| Hu Fc Block Pure Fc1.3216 | Cat# 564220 | BD Pharmingen |
| Hu IFN-Gma BV421 B27 | Cat# 562988 | BD Pharmingen |
| Hu Solbl Ptein CBA Buf Kit | Cat# 558264 | BD Pharmingen |
| Hu TNF CBA Flex Set D9 | Cat# 558273 | BD Pharmingen |
| Hu/NHP TNF APC MAb11 | Cat# 551384 | BD Pharmingen |
| Hu CD86 PerCP-Cy5.5 2331 (FUN-1) | Cat# 561129 | BD Pharmingen |
| Ms CD3e APC-Cy7 145-2C11 | Cat# 557596 | BD Pharmingen |
| Ms CD137 BV650 1AH2 | Cat# 740499 | BD Pharmingen |
| ***Continued*** |  |  |
| **REAGENT or RESOURCE** | **IDENTIFIER** | **SOURCE** |
| Ms CD152 APC-R700 UC10-4F10-11 | Cat# 565778 | BD Pharmingen |
| Ms CD16/CD32 Pure 2.4G2 | Cat# 553141 | BD Pharmingen |
| Ms CD206 Alexa 647 MR5D3 | Cat# 565250 | BD Pharmingen |
| Ms CD279 APC J43 | Cat# 562671 | BD Pharmingen |
| Ms CD4 PerCP-Cy5.5 RM4-5 | Cat# 550954 | BD Pharmingen |
| Ms CD44 PE IM7 | Cat# 553134 | BD Pharmingen |
| Ms CD45 FITC 30-F11 | Cat# 553079 | BD Pharmingen |
| Ms CD62L BUV395 MEL-14 | Cat# 740218 | BD Pharmingen |
| Ms CD69 PE-Cy7 H1.2F3 | Cat# 552879 | BD Pharmingen |
| Ms CD80 BV650 16-10A1 | Cat# 563687 | BD Pharmingen |
| Ms CD86 BB700 GL1 | Cat# 742120 | BD Pharmingen |
| Ms CD8a BV786 53-6.7 | Cat# 563332 | BD Pharmingen |
| Ms F4/80 BV421 T45-2342 | Cat# 565411 | BD Pharmingen |
| Ms IFN-Gma BV421 XMG1.2 | Cat# 563376 | BD Pharmingen |
| Ms Ig Kpa Comp Bead Set | Cat# 552843 | BD Pharmingen |
| Ms TNF PE MP6-XT22 | Cat# 554419 | BD Pharmingen |
| Ms/Rat Solbl Ptein CBA Buf Kit | Cat# 558266 | BD Pharmingen |
| Anti-Human CD3 FITC | Cat# 11-0039-42 | eBioscience |
| Anti-Human HLA-DR PerCP-Cyanine5.5 | Cat# 45-9956-42 | eBioscience |
| Anti-Human CD274 (PD-L1, B7-H1) PE-Cyanine7 | Cat# 25-5983-42 | eBioscience |
| Anti-Mouse CD163 Monoclonal Antibody (TNKUPJ) PE-Cyanine7 | Cat# 25-1631-82 | eBioscience |
| Anti-Mouse CD11b Monoclonal Antibody (M1/70) APC-eFluor™ 780 | Cat# 47-0112-82 | eBioscience |
| Anti-Mouse CD3e Monoclonal Antibody (145-2C11), APC-eFluor™ 780 | Cat# 47-0031-82 | eBioscience |
| Anti-Mouse CD4 Monoclonal Antibody (RM4-5), FITC | Cat# 11-0042-82 | eBioscience |
| Anti-Mouse CD45 Monoclonal Antibody (30-F11), Alexa Fluor™ 700 | Cat# 56-0451-82 | eBioscience |
| Anti-Mouse CD45 Monoclonal Antibody (30-F11), FITC | Cat# 11-0451-82 | eBioscience |
| Anti-Mouse CD62L (L-Selectin) Monoclonal Antibody (MEL-14), PE | Cat# 12-0621-82 | eBioscience |
| Anti-Mouse CD8a Monoclonal Antibody (53-6.7), PerCP-Cyanine5.5 | Cat# 45-0081-82 | eBioscience |
| Anti-Mouse F4/80 Monoclonal Antibody (BM8), PE | Cat# 12-4801-82 | eBioscience |
| Anti-Mouse MHC Class II (I-A/I-E) Monoclonal Antibody (M5/114.15.2), FITC | Cat# 11-5321-81 | eBioscience |
| Anti-Mouse TNF alpha Monoclonal Antibody (MP6-XT22), PE | Cat# 12-7321-82 | eBioscience |
| Anti-Mouse CD206 (MMR) Monoclonal Antibody (MR6F3), APC | Cat# 17-2061-82 | eBioscience |
| Alexa Fluor® 700 anti-mouse/human CD11b M1/70 | Cat# 101222 | biolegend |
| APC anti-human TNF-α | Cat# 502912 | biolegend |
| Cell activation coktail (with brefekldin A) | Cat# 423303 | Biolegend |
| Brilliant Violet 421™ anti-mouse IFN-γ | Cat# 505829 | biolegend |
| PE/Dazzle 594 anti-human/mouse Granzyme B Recombinant | Cat# 372216 | biolegend |

**Supplementary Table S2. The drug administration for *in vivo* experiments**

| **Reagents** | **Administration** | **Dosage** | **Dosing frequency** |
| --- | --- | --- | --- |
| APG-2575 | Oral | 50 mg/kg | Once a day |
| Pembrolizumab | Intraperitoneal injection | 10 mg/kg | Twice a week |
| Anti-PD-1 mouse antibody | Intraperitoneal injection | 10 mg/kg | Twice a week |
| Anti-PD-1 mouse IgG1 | Intraperitoneal injection | 10 mg/kg | Twice a week |
| Anti-CD3 neutralizing antibody | Intraperitoneal injection | 300 μg per mice | Every 4 days |
| Anti-CD4 neutralizing antibody | Intraperitoneal injection | 200 μg per mice | Every 4 days |
| Anti-CD8 neutralizing antibody | Intraperitoneal injection | 200 μg per mice | Every 4 days |
| Anti-CCL5 neutralizing antibody | Intraperitoneal injection | 100 μg per mice | Once a day |
| Anti-CXCL0 neutralizing antibody | Intraperitoneal injection | 200 μg per mice | Every 2 days |
| PLX3397 | Oral | 100 mg/kg | Once a day |
| Isotype control | Intraperitoneal injection | 100 μl per mice | Every 4 days |
| JSH-23 | Intraperitoneal injection | 5 mg/kg | Every 2 days |
| INF39 | Oral | 25 mg/kg | Once a day |

**Supplementary Table S3. qRT-PCR primer sequences.**

| **Gene** | **Forward** | **Reverse** |
| --- | --- | --- |
| *hGAPDH* | CGCTGAGTACGTCGTGGAGTC | GCTGATGATCTTGAGGCTGTTGTC |
| *hIL-1β* | ATGATGGCTTATTACAGTGGCAA | GTCGGAGATTCGTAGCTGGA |
| *hNOS2* | TTCAGTATCACAACCTCAGCAAG | TGGACCTGCAAGTTAAAATCCC |
| *hIFN-γ* | TCGGTAACTGACTTGAATGTCCA | TCGCTTCCCTGTTTTAGCTGC |
| *hTNF-α* | CCTCTCTCTAATCAGCCCTCTG | GAGGACCTGGGAGTAGATGAG |
| *hARG-1* | GTGGAAACTTGCATGGACAAC | AATCCTGGCACATCGGGAATC |
| *hMRC-1* | TCCGGGTGCTGTTCTCCTA | CCAGTCTGTTTTTGATGGCACT |
| *hTGF-β* | GGCCAGATCCTGTCCAAGC | GTGGGTTTCCACCATTAGCAC |
| *hIL-10* | GACTTTAAGGGTTACCTGGGTTG | TCACATGCGCCTTGATGTCTG |
| *hNLRP3* | GATCTTCGCTGCGATCAACAG | CGTGCATTATCTGAACCCCAC |
| *hCCL5* | CCAGCAGTCGTCTTTGTCAC | CTCTGGGTTGGCACACACTT |
| *hCXCL10* | GTGGCATTCAAGGAGTACCTC | TGATGGCCTTCGATTCTGGATT |
| *mGapdh* | AGGTCGGTGTGAACGGATTTG | TGTAGACCATGTAGTTGAGGTCA |
| *mIl-1β* | GCAACTGTTCCTGAACTCAACT | ATCTTTTGGGGTCCGTCAACT |
| *mNos2* | GTTCTCAGCCCAACAATACAAGA | GTGGACGGGTCGATGTCAC |
| *mIfn-γ* | GAGCCAGATTATCTCTTTCTACCT | GTTGTTGACCTCAAACTTGGC |
| *mTnf-α* | CCCACGTCGTAGCAAACCAC | GCAGCCTTGTCCCTTGAA |
| *mArg-1* | CTGAGAGATTCAAGGCAAGAGG | GAACGCGCTATCTTACCCCAG |
| *mMrc-1* | CTCTGTTCAGCTATTGGACGC | CGGAATTTCTGGGATTCAGCTTC |
| *mTgf-β* | AGCTGCTTATCCCAGATTCAGCCA | TATCGAGGCCAGCTTGTTTGAGGA |
| *mIl-10* | GGTTGCCAAGCCTTATCGGA | ACCTGCTCCACTGCCTTGCT |
| *mNlrp3* | ATTACCCGCCCGAGAAAGG | TCGCAGCAAAGATCCACACAG |
| *mCcl5* | GCTGCTTTGCCTACCTCTCC | TCGAGTGACAAACACGACTGC |
| *mCxcl10* | CCAAGTGCTGCCGTCATTTTC | GGCTCGCAGGGATGATTTCAA |

**Supplementary Table S4. The probe sequences utilized in EMSA.**

| **Probe** | **Sequence** |
| --- | --- |
| NF-κB EMSA probe Biotin labeled | GGAAAATCCATAAAAAGGGAACCCCCG |
|  | CGGGGGTTCCCTTTTTATGGATTTTCC |
| NF-κB EMSA probe No labeled | GGAAAATCCATAAAAAGGGAACCCCCG |
|  | CGGGGGTTCCCTTTTTATGGATTTTCC |
| NF-κB EMSA probe site 1 | -1303nt to -1292nt (AGGGAACCCCCG) |
| NF-κB EMSA probe site 2 | -1238nt to -1228nt (GGAAAATCCAT) |
| NF-κB EMSA probe Mutation Biotin labeled | GGAGGATCCATAAAAAGGGAACCAACG |
|  | CGTTGGTTCCCTTTTTATGGATCCTCC |

**Supplementary Table S5**. **Cell-Type-Specific Gene Markers obtained from the Literature.**

| **Gene** | **Cell Type** | **Reference** |
| --- | --- | --- |
| *CD79A* | B cells | Dong Y et al., 2022 |
| *MS4A1* | B cells | Mattiola I et al., 2021 |
| *CD79B* | B cells | Mandato E et al., 2023 |
| *CD3D* | CD4+ T cells | Limbach M et al., 2016 |
| *CD3E* | CD4+ T cells | Limbach M et al., 2016 |
| *CD4* | CD4+ T cells | Teghanemt A et al., 2022 |
| *CD3D* | CD8+ T cells | Zhu Z et al., 2021 |
| *CD3E* | CD8+ T cells | Zheng X et al., 2021 |
| *CD8A* | CD8+ T cells | Zhao Z et al., 2022 |
| *CD8B* | CD8+ T cells | Sun R et al., 2018 |
| *FSCN1* | DC | Zernecke A et al., 2023 |
| *LAMP3* | DC | Zhang Q et al., 2019 |
| *CCR7* | DC | Donnelly H et al., 2023 |
| *CD68* | Macrophages/Monocytes | Rebelo SP et al., 2018 |
| *MS4A6A* | Macrophages/Monocytes | Zhang C et al., 2022 |
| *TUBB6* | Macrophages/Monocytes | Sun QY et al., 2023 |
| *BEST1* | Macrophages/Monocytes | Zhang L et al., 2023 |
| *XCL1* | NK cells | Woo YD et al., 2018 |
| *XCL2* | NK cells | de Andrade LF et al., 2019 |
| *TRDC* | NK cells | Halloran PF et al., 2022 |

**Supplementary Table S6. Characteristics of all patients (n=23) (100%).**

| **Characteristics** | **Cases (n=23)** | **Percentage (%)** |
| --- | --- | --- |
| **Age (years)** |  |  |
| Median (range) | 63 (28-75) |  |
| ＜60 | 10 | 43.5 |
| ≥60 | 13 | 56.5 |
| **Gender** |  |  |
| Male | 16 | 69.6 |
| Female | 7 | 30.4 |
| **Smoking history** |  |  |
| Non-smoker | 12 | 52.2 |
| Smoker | 11 | 47.8 |
| **Drinking history** |  |  |
| Yes | 17 | 63.2 |
| No | 6 | 36.8 |
| **Family history** |  |  |
| Yes | 4 | 17.4 |
| No | 19 | 82.6 |
| **Pathological type** |  |  |
| Adenocarcinoma | 12 | 52.2 |
| Non-adenocarcinoma | 11 | 47.8 |
| **T-stage** |  |  |
| T1 | 6 | 26.1 |
| T2 | 2 | 8.7 |
| T3 | 3 | 13 |
| T4 | 12 | 52.2 |
| **N-stage** |  |  |
| N1 | 3 | 13 |
| N2 | 5 | 21.7 |
| N3 | 15 | 65.3 |
| **M-stage** |  |  |
| M1a | 7 | 30.4 |
| M1b | 6 | 26.1 |
| M1c | 10 | 43.5 |
| **Disease stage** |  |  |
| IVa | 7 | 30.4 |
| IVb | 16 | 69.6 |
| **Radiotherapy** |  |  |
| No | 16 | 69.6 |
| Yes | 7 | 30.4 |
| **Timepoint of histology** |  |  |
| Baseline | 23 | 100 |
| **Type of immunotherapy** |  |  |
| KN046（CTLA-4×PD-L1） | 23 | 100 |
| Administration dose |  |  |
| 3mg/kg | 10 | 43.5 |
| 5mg/kg | 8 | 34.8 |
| 300mg/once | 5 | 21.7 |
| Administration cycle |  |  |
| Every 3 weeks | 23 | 100 |
| Mode of administration |  |  |
| Intravenous drip | 23 | 100 |

**Supplementary Figures**

**
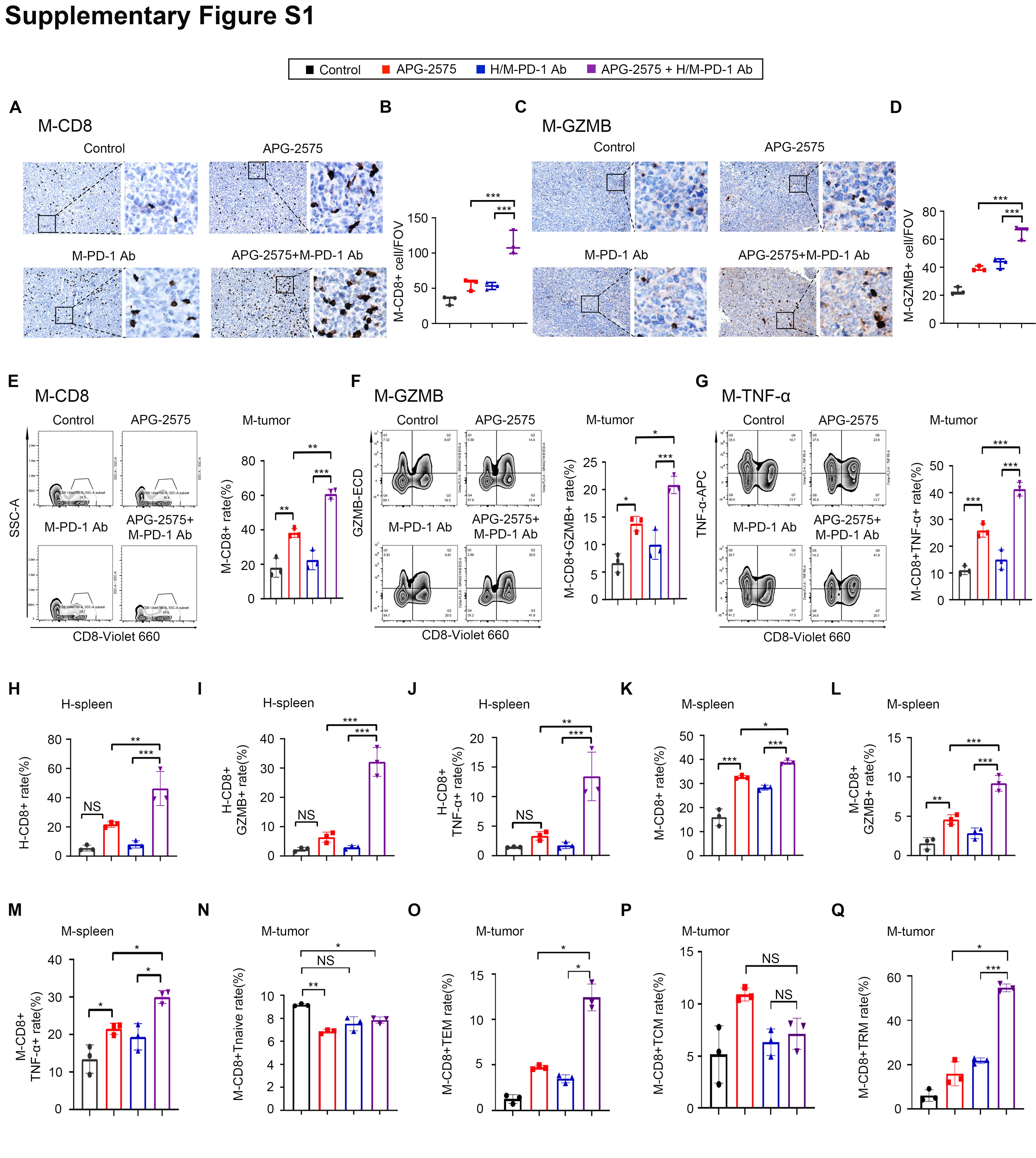
**

**Supplementary Figure S1: APG-2575 potentiates the efficacy of an immune checkpoint inhibitor in humanized CD34+ models and C57BL/6 mice models.**

(A-D) Representative IHC staining of CD8 and GZMB in C57BL/6 xenograft tumors under various treatments. (E-G) Flow cytometry analysis of CD8+, CD8+GZMB+ and CD8+TNF-α+ T cells in C57BL/6 xenograft tumors from different treatments. (H-J) Flow cytometry analysis of splenic CD8+, CD8+GZMB+ and CD8+TNF-α+ T cells in humanized CD34+ models from different treatments. (K-M) Flow cytometry analysis of splenic CD8+, CD8+GZMB+ and CD8+TNF-α+ T cells in C57BL/6 mice from different treatments. (N-Q) Tumor-bearing mice with different treatments. The proportion of naive-like CD8+ T cells, effector memory CD8+ T cells (TEM), central memory CD8+ T cells (TCM) and tissue-resident memory CD8+ T cells (TRM) in C57BL/6 mice as quantified by flow cytometry.

**
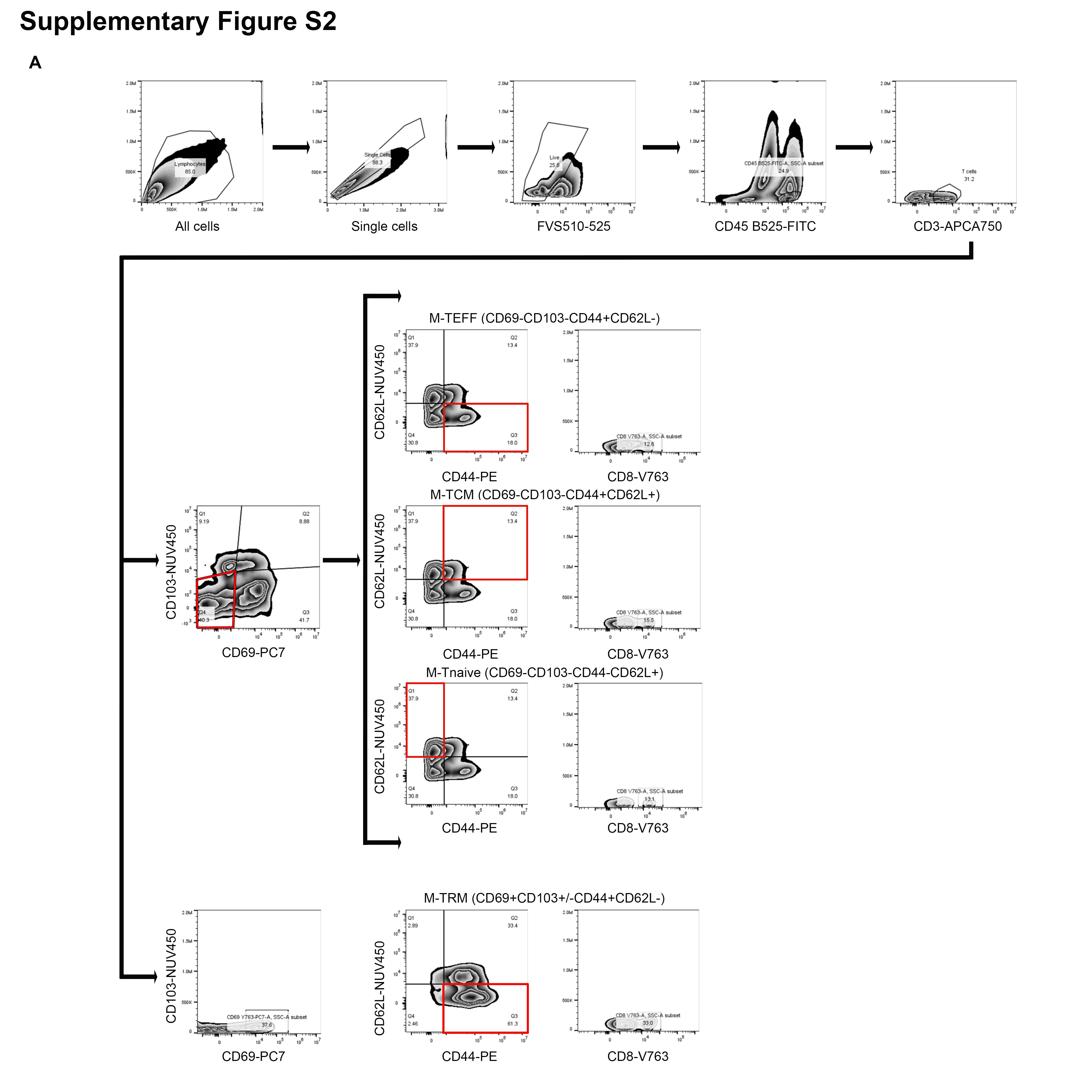
**

**Supplementary Figure S2: (A) The gating strategy for the CD8+ T cell subpopulations of tumor-infiltrating lymphocytes in C57BL/6 mice as measured by flow cytometry analysis.**


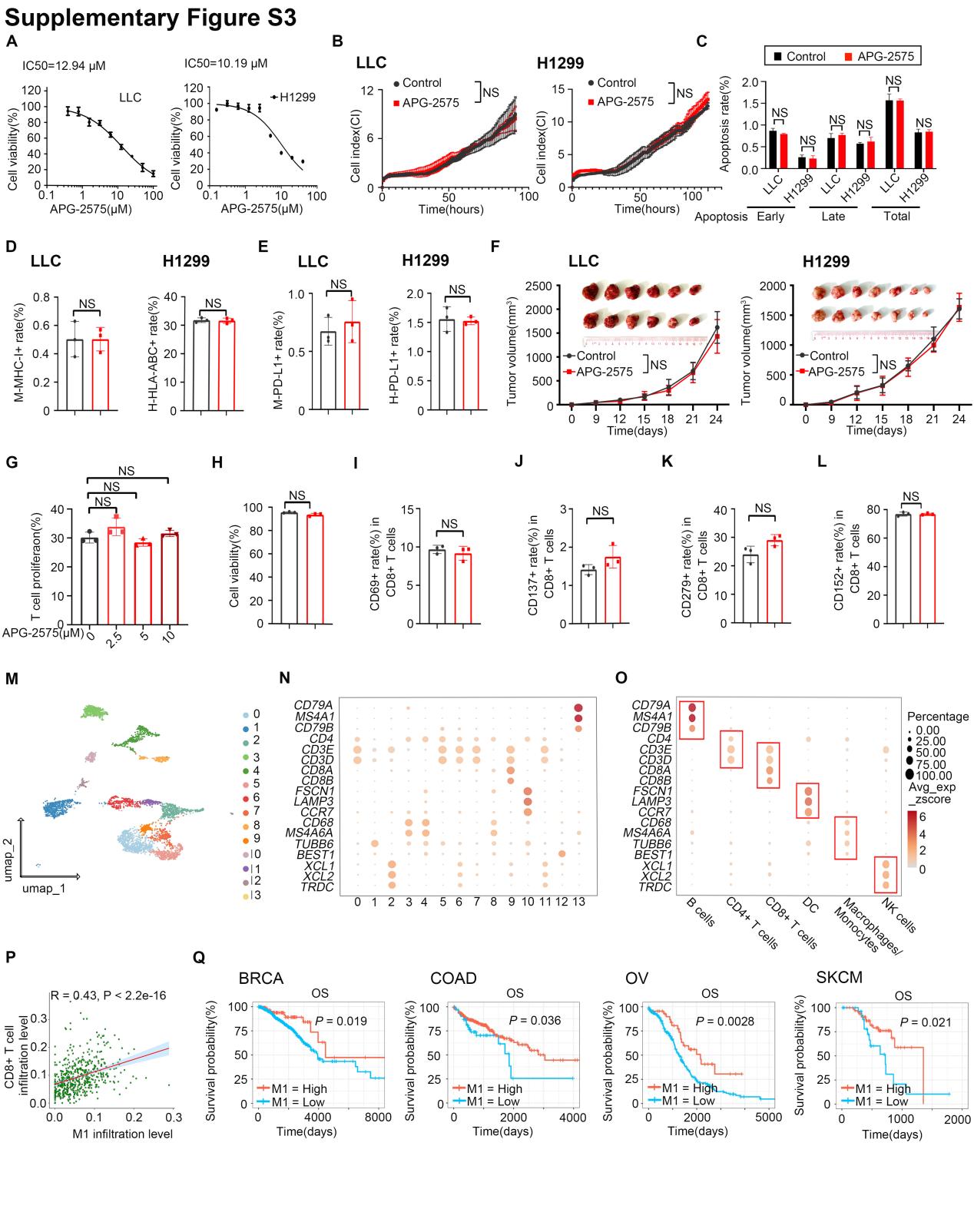


**Supplementary Figure S3: The anti-tumor activity of APG-2575 is CD8+ T cell driven and the efficacy of immunotherapy is positively correlated with M1-like macrophages while negatively correlated with M2-like macrophages.**

(A) *In vitro* response of a murine lung cancer LLC cell line and human H1299 cell line to APG-2575. Cell viability following 72 hours of APG-2575 treatment. (B) Proliferation as measured during the course of 72 hours treatment with APG-2575 in RTCA assay. (C) Percentage of apoptosis was detected in LLC and H1299 cells with or without APG-2575 treatment. (D-E) Cell surface MHC-I and PD-L1 expression in the absence or presence of APG-2575 treatment. (F) Tumor volume (mm^3^) plots of nude mice with established LLC tumors or H1299 tumors treated with APG-2575 alone or control. (G) Splenic CD8+ T cells were labelled with carboxy fluorescein succinimidyl ester (CFSE) and then stimulated with CD3/CD28 beads and IL-2 in the presence of 0μM, 2.5μM, 5μM, and 10μM APG-2575 for 72 hours. Cell proliferation was measured by the quantification of T cell proliferation. (H-L) T cell viability (Annexin V and PI negative cells), T cell activation (CD69, CD137), and T cell anergy (CD279, CD152) of splenic CD8+ T cells with or without APG-2575 treatment were analyzed. (M) UMAP plot of human tumor-infiltrating CD45+ cells from two groups merged analyzed by scRNA-seq. (N-O) Dot plot depicting the average scaled gene expression of selected marker genes for each cell cluster (N) or each annotated cell type (O). Dot size corresponds to the percentage of cells expressing each gene. (P) Scatterplot results from the Pearson correlation analysis of M1 macrophage infiltration levels and the infiltration of CD8+ T cells in the TCGA cohorts. (Q) Kaplan-Meier OS analysis of patients based on M1 macrophage infiltration levels in various cancer types based on TCGA cohorts.


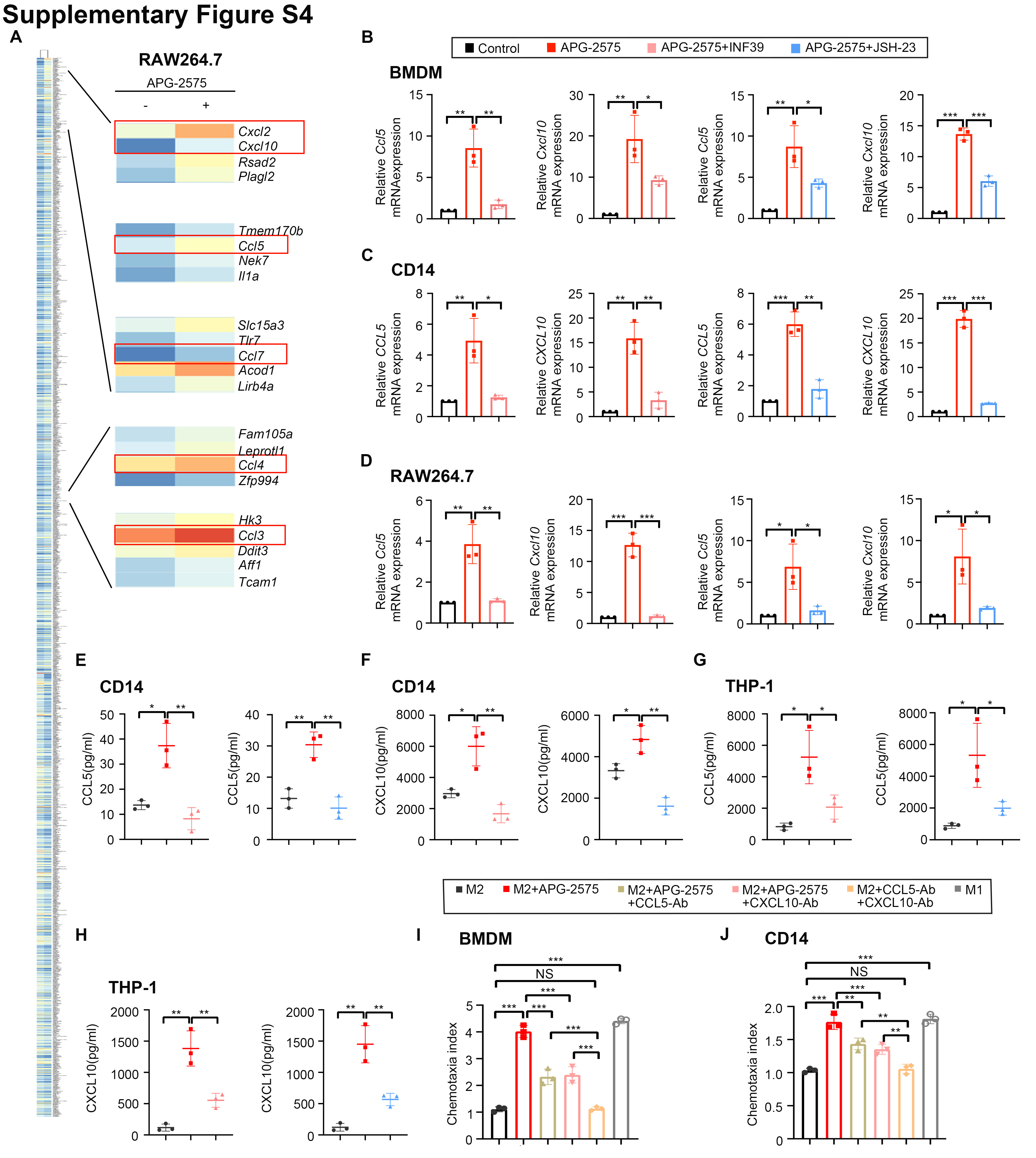


**Supplementary Figure S4: APG-2575 resets M2 macrophages to the M1 phenotype, promoting CD8+ T cells infiltration via CCL5 and CXCL10.**

(A) IL-4-activated RAW264.7 cells treated with APG-2575 or control, respectively, subjected to RNA-seq analysis. (B-D) The mRNA expression of *CCL5* and *CXCL10* in various IL-4-activated macrophages treated with or without APG-2575 in the presence or absence of INF39 or JSH-23. (E-F) IL-4-activated CD14+ monocytes-derived macrophages treated with or without APG-2575 in the presence or absence of JSH-23 or INF39 for 24 hours. The CCL5 and CXCL10 in the supernatants as detected by CBA. (G-H) IL-4-activated THP-1-originated macrophages treated with or without APG-2575 in the presence or absence of JSH-23 or INF39 for 24 hours. The CCL5 and CXCL10 in the supernatants as detected by CBA. (I) Chemotaxis of CD8+ T cells towards BMDM-M1 as well as BMDM-M2 treated with APG-2575 alone, APG-2575 plus CCL5 and/or CXCL10-neutralizing antibodies. (J) Chemotaxis of CD8+ T cells towards CD14+ monocytes-derived macrophages-M1 as well as CD14+ monocytes-derived macrophages-M2 treated with APG-2575 alone, APG-2575 plus CCL5 and/or CXCL10-neutralizing antibodies.

**
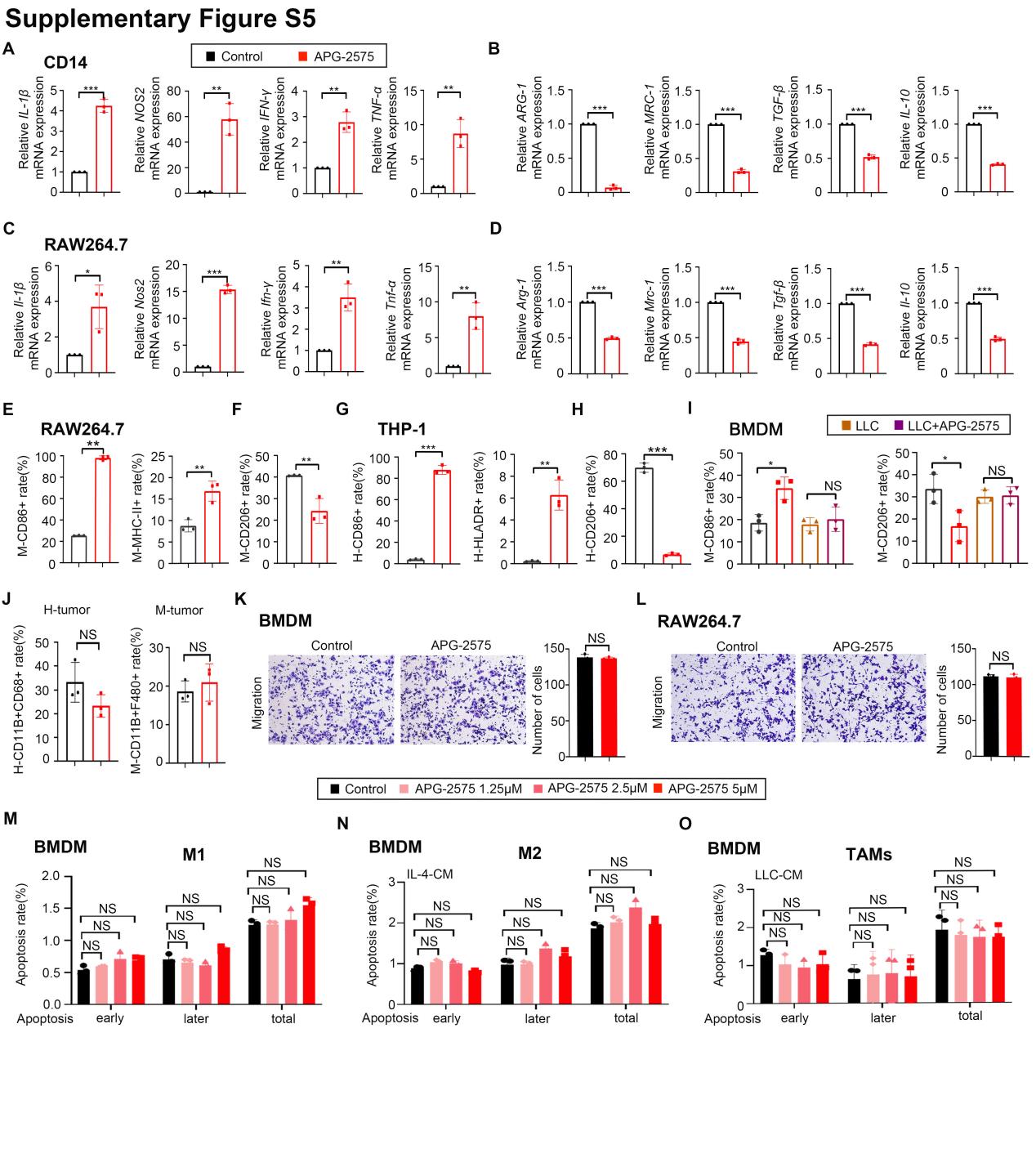
**

**Supplementary Figure S5: APG-2575 effectively repolarized M2-like macrophages to the M1 phenotype .**

(A-D) The mRNA expressions of M1/M2 related markers in IL-4-activated macrophages with or without APG-2575 treatment. (E-F) The quantification of CD86, MHC-II and CD206 in IL-4-activated RAW264.7 cells with or without APG-2575 treatment. (G-H) The quantification of CD86, HLA-DR and CD206 in IL-4-activated THP-1-originated macrophages with or without APG-2575 treatment. (I) LLC cells with or without APG-2575 treatment co-cultured with IL-4-activated BMDM cells. The expression of CD86 and CD206 as analyzed by flow cytometry. (J) Flow cytometry analysis of CD11B+CD68+ and CD11B+F4/80+ total macrophages in tumor tissues after PBS or APG-2575 treatment in humanized CD34+ models and C57BL/6 mice. (K-L) Representative pictures exhibited migration of BMDM/RAW264.7 cells detected by microscope and the quantity of migratory BMDM/RAW264.7 cells per membrane was observed by utilizing a microscope. (M-O) Percentage of apoptosis was detected in M1 macrophages, M2 macrophages ana TCM conditioned macrophages with 0μM, 1.25μM, 2.5μM, 5μM APG-2575 treatment.

**
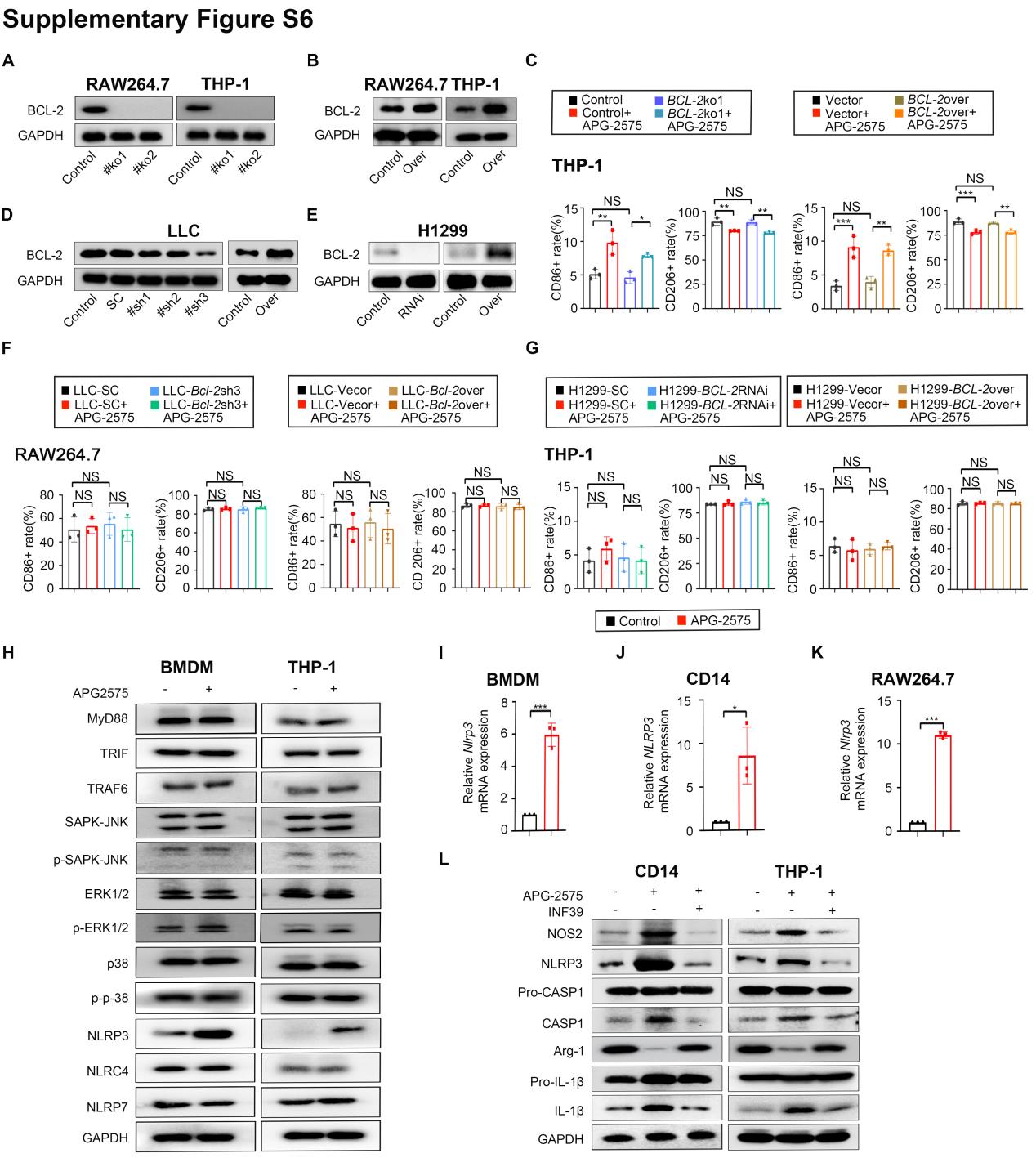
**

**Supplementary Figure S6: *BCL-2* gene downregulation or upregulation in macrophages or cancer cells have no effect on APG-2575-induced M1 macrophage polarization.**

(A) Western blotting analysis for the BCL-2 protein expression in the control and *BCL-2*-knockout RAW264.7 and THP-1 cells. (B) Western blotting analysis for the BCL-2 protein expression in the control and *BCL-2*-overexpression RAW264.7 and THP-1 cells. (C) The quantification of CD86 and CD206 in IL-4-activated *BCL-2*-knockout or overexpression THP-1 cells treated with APG-2575 or control. (D) Western blotting analysis for the BCL-2 protein expression in the control and *Bcl-2*-knockdown or overexpression LLC cells. (E) Western blotting analysis for the BCL-2 protein expression in the control and *BCL-2*-RNAi or overexpression H1299 cells. (F) *Bcl-2*-knockdown or overexpression LLC cells with or without APG-2575 treatment co-cultured with IL-4-activated RAW264.7 cells. The expression of CD86 and CD206 of RAW264.7 cells were analyzed. (G) *BCL-2*-RNAi or overexpression of H1299 cells with or without APG-2575 treatment co-cultured with IL-4-activated THP-1-originated macrophages. The expression of CD86 and CD206 of THP-1 cells were analyzed. (H) Western blotting analysis of TLRs-MyD88-TRIF signaling, MAPK pathways, NLRP3, NLRC4 and NLRP7 of IL-4-activated macrophages cultured with APG-2575 or control for 24 hours. (I-K) The mRNA expressions of *Nlrp3* in IL-4-activated macrophages with or without APG-2575 treatment. (L) Western blotting analysis of NOS2, NLRP3, caspase-1, Arg-1, IL-1β of IL-4-activated macrophages cultured with APG-2575 in the presence or absence of INF39 for 24 hours.

**
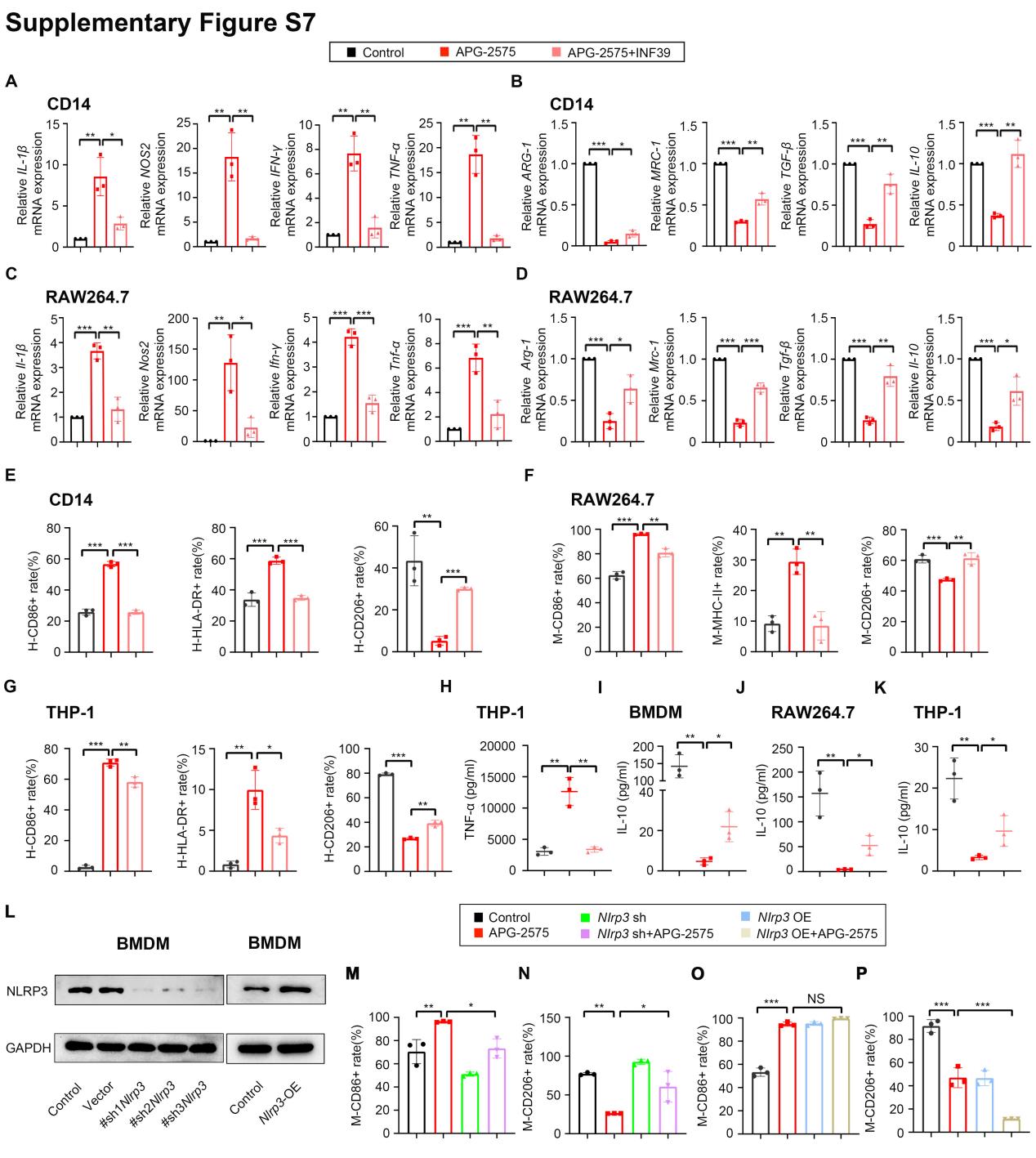
**

**Supplementary Figure S7: APG-2575 enhanced M1 polarization via NLRP3 inflammasome activation.** (A-D) The mRNA expressions of M1/M2 related markers in IL-4-activated macrophages treated with APG-2575 in the presence or absence of INF39 for 24 hours. (E) The quantification of CD86, HLA-DR and CD206 in IL-4-activated CD14+ monocytes-derived macrophages treated with APG-2575 in the presence or absence of INF39 for 24 hours. (F) The quantification of CD86, MHC-II and CD206 in IL-4-activated RAW264.7 cells treated with APG-2575 in the presence or absence of INF39 for 24 hours. (G) The quantification of CD86, HLA-DR and CD206 in IL-4-activated THP-1-originated macrophages treated with APG-2575 in the presence or absence of INF39 for 24 hours. (H) IL-4-activated THP-1-originated macrophages treated with or without APG-2575 in the presence or absence of INF39 for 24 hours. The TNF-α in the supernatants as detected by CBA. (I-K) IL-4-activated macrophages were treated with or without APG-2575 in the presence or absence of INF39 for 24 hours. IL-10 in the supernatants as measured by ELISA. (L) Western blotting identified stable *Nlrp3* knockdown and overexpression in BMDM cells. (M-P) Flow cytometry analysis of CD86 and CD206 of stable *Nlrp3* knockdown and overexpression macrophages cells treated with APG-2575 or control.

**
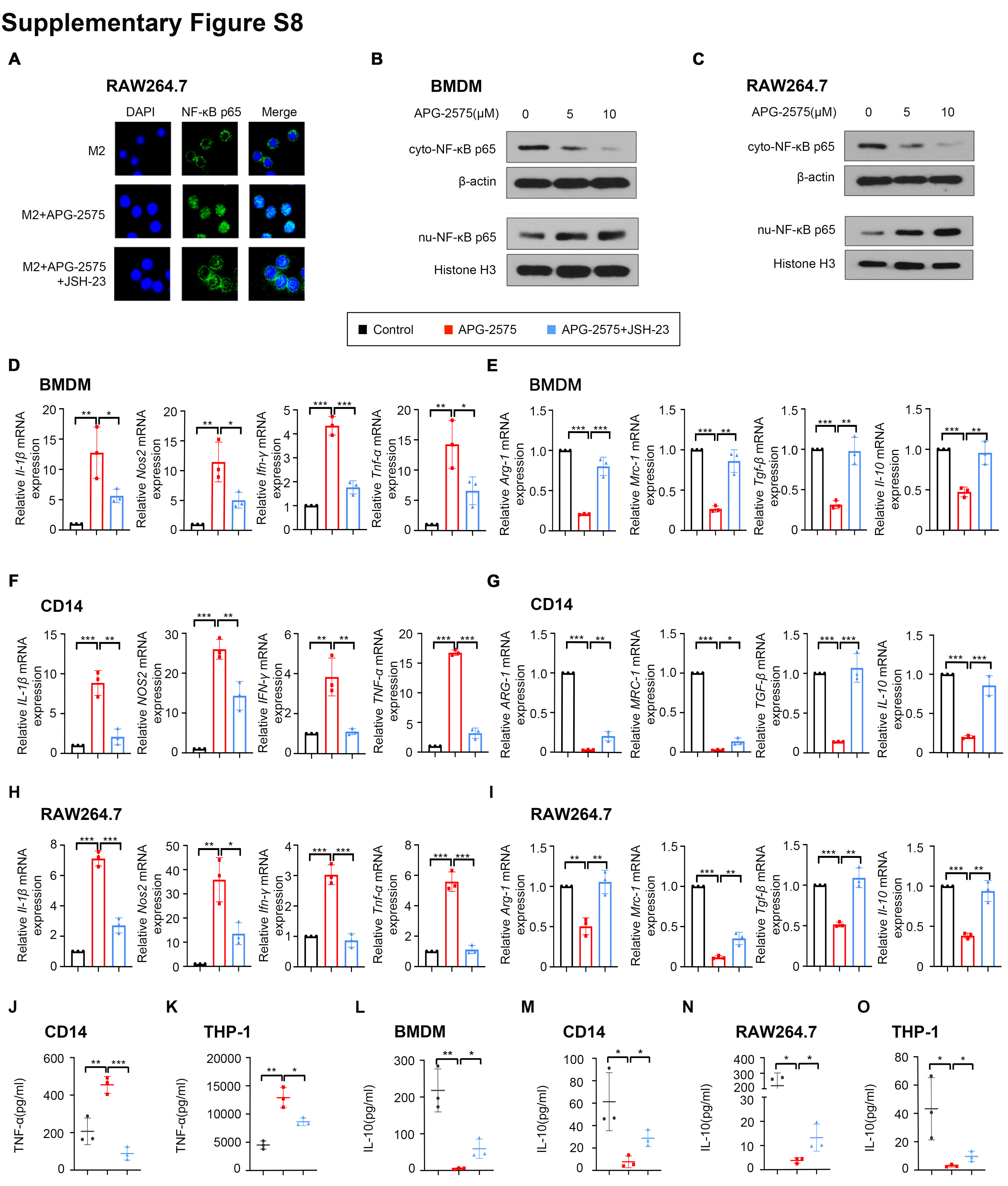
**

**Supplementary Figure S8: APG-2575 induced NLRP3 transcription and expression via enhancing NF-κB** **nuclear localization.**

(A) NF-κB p65 in IL-4-activated RAW264.7 cells after APG-2575 treatment with JSH-23 as examined using a confocal fluorescent microscope. Green, NF-κB p65; blue, DAPI. Scale bar, 20 μm. (B-C) Protein expressions of NF-κB p65 in the cytoplasm (Cyto) or nucleus (Nuc) of IL-4-activated BMDM and Raw264.7 cells as measured by western blotting. (D-I) The mRNA expressions of M1/M2 related markers in IL-4-activated macrophages treated with APG-2575 in the presence or absence of JSH-23 for 24 hours. (J-K) IL-4-activated macrophages treated with or without APG-2575 in the presence or absence of JSH-23 for 24 hours. The TNF-α in the supernatants as detected by CBA. (L-O) IL-4-activated macrophages treated with or without APG-2575 in the presence or absence of JSH-23 for 24 hours. The IL-10 in the supernatants as measured by ELISA.

**
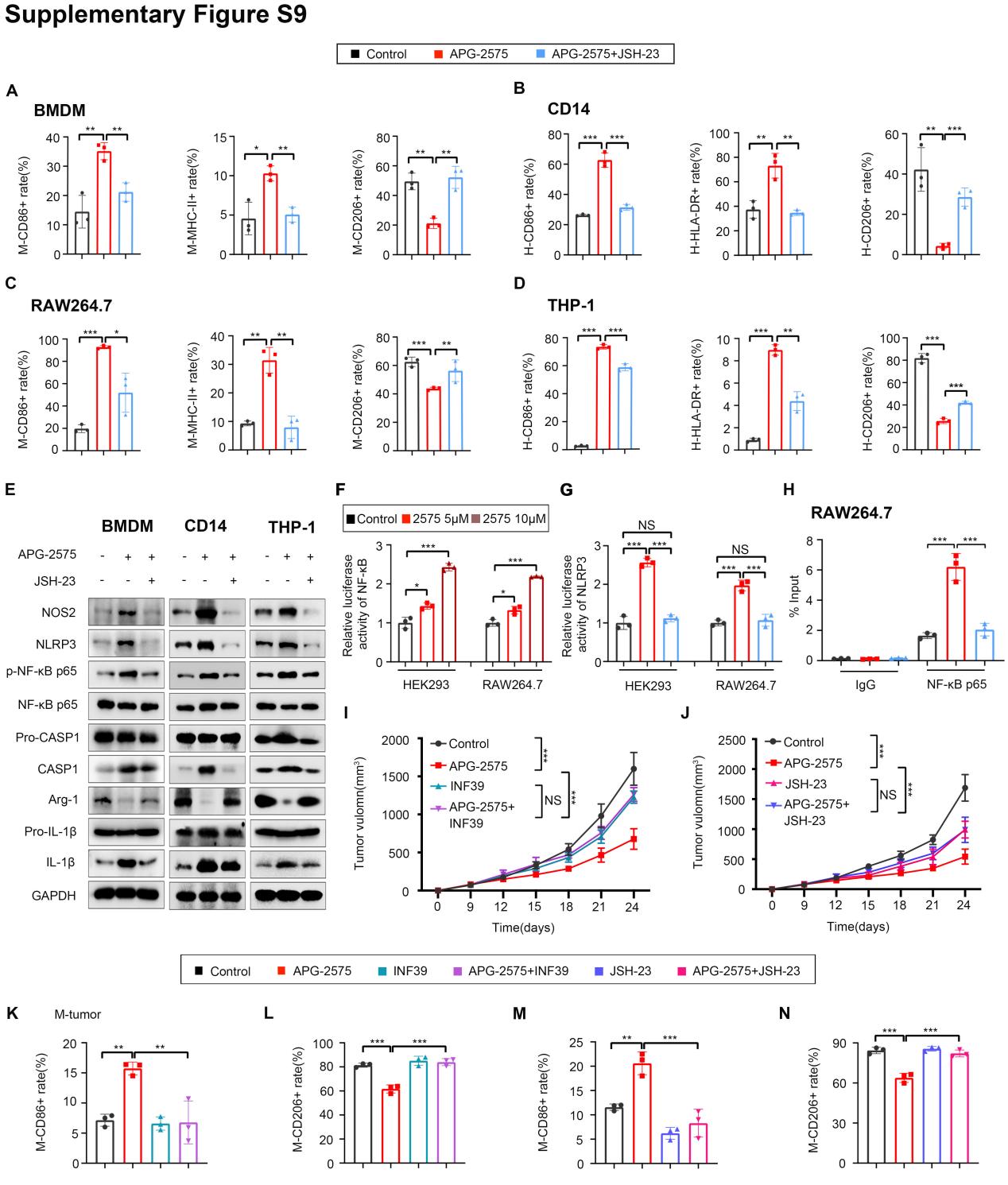
**

**Supplementary Figure S9: APG-2575 enhances M1 polarization via activating the NF-κB/NLRP3 signaling pathway *in vitro* and *in vivo*.**

(A, C) The quantification of CD86, MHC-II and CD206 in IL-4-activated BMDM and IL-4-activated RAW264.7 cells treated with APG-2575 in the presence or absence of JSH-23 for 24 hours. (B, D) The quantification of CD86, HLA-DR and CD206 in IL-4-activated CD14+ monocytes-derived macrophages and IL-4-activated THP-1-originated macrophages treated with APG-2575 in the presence or absence of JSH-23 for 24 hours. (E) Western blotting analysis of NOS2, NLRP3, caspase-1, Arg-1, IL-1β of IL-4-activated macrophages treated with APG-2575 in the presence or absence of JSH-23 for 24 hours. (F) Luciferase reporter assays with HEK293 and IL-4-activated Raw264.7 cells. (G) Luciferase reporter assays showing that APG-2575 upregulates *Nlrp3* transcription in HEK293 and IL-4-activated Raw264.7 cells, which can be inhibited by JSH23. (H) ChIP assay, showing the recruitment of NF-κB p65 to the Nlrp3 promoter in IL-4-activated RAW264.7 cells. IL-4-activated RAW264.7 cells treated with APG-2575 in the presence or absence of JSH-23 for 24 hours. (I-J) Tumor volumes of C57BL/6 mice as determined on the indicated day under various treatments. (K-N) Flow cytometry analysis of CD86 and CD206 in TAMs after the indicated treatments (control, APG-2575, INF39, JSH-23, APG-2575+INF39 and APG-2575+JSH-23) in C57BL/6 mice bearing a subcutaneous LLC tumor.

**
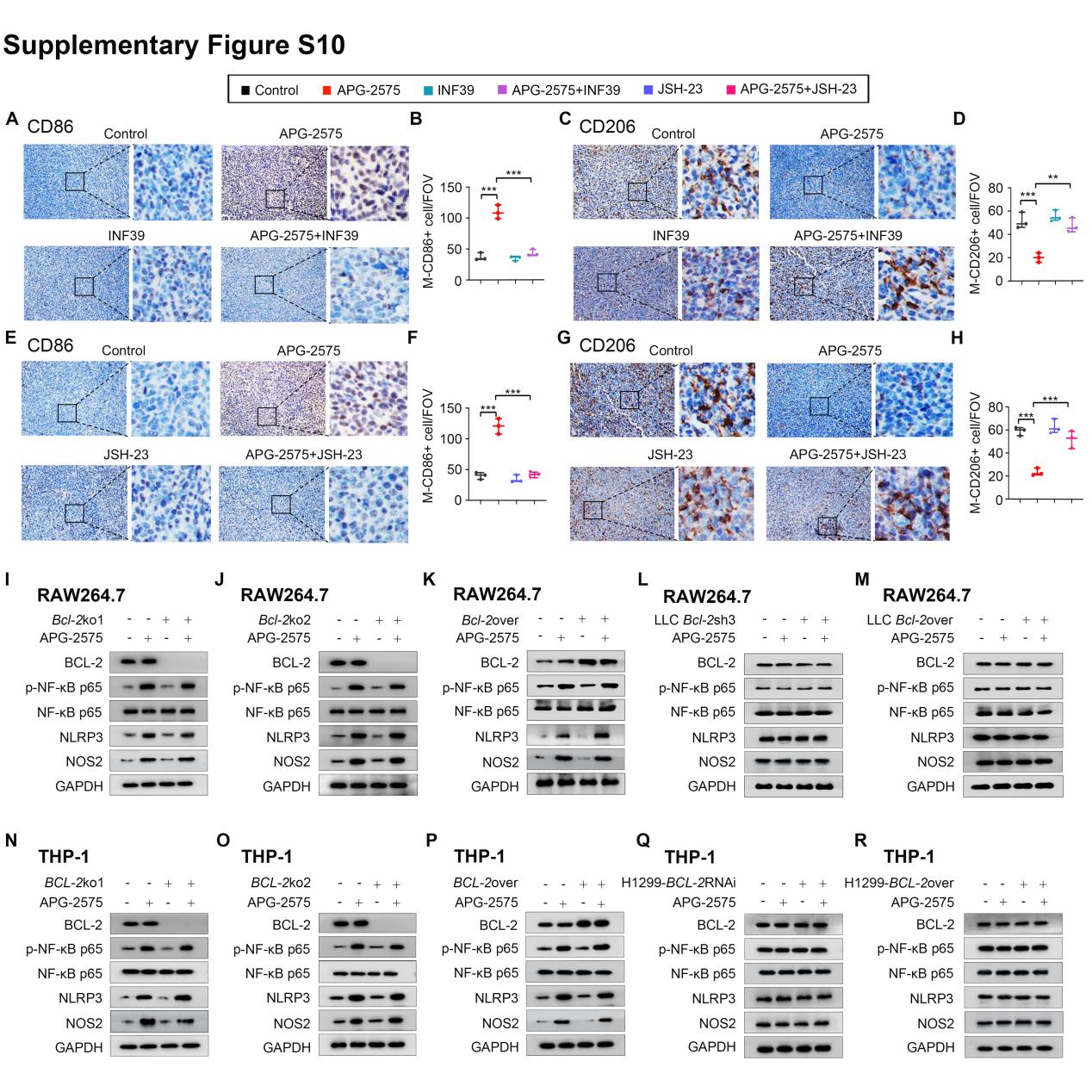
**

**Supplementary Figure S10: *BCL-2* gene alteration in macrophages or cancer cells had no impact on APG-2575-mediated activation of** **the NF-κB/NLRP3 signaling pathway.**

(A-H) IHC staining analysis of CD86 and CD206 in TAMs after the indicated treatments (control, APG-2575, INF39, JSH-23, APG-2575+INF39 and APG-2575+JSH-23) in C57BL/6 mice bearing a subcutaneous LLC tumor. (I-K) Western blotting analysis of the phosphorylation levels of NF-κB p65 of IL-4-activated *Bcl-2* knockout and overexpression RAW264.7 cells treated with APG-2575 or control. (L-M) *Bcl-2*-knockdown or overexpression LLC cells with or without APG-2575 treatment co-cultured with IL-4-activated RAW264.7 cells. The phosphorylation expression levels of NF-κB p65 of IL-4-activated RAW264.7 cells were analyzed. (N-P) Western blotting analysis of the phosphorylation levels of NF-κB p65 of *BCL-2* knockout and overexpression IL-4-activated THP-1-originated macrophages treated with APG-2575 or control. (Q-R) *BCL-2*-RNAi or overexpression of H1299 cells with or without APG-2575 treatment co-cultured with IL-4-activated THP-1-originated macrophages. The phosphorylation expression levels of NF-κB p65 of IL-4-activated THP-1-originated macrophages were analyzed.

**
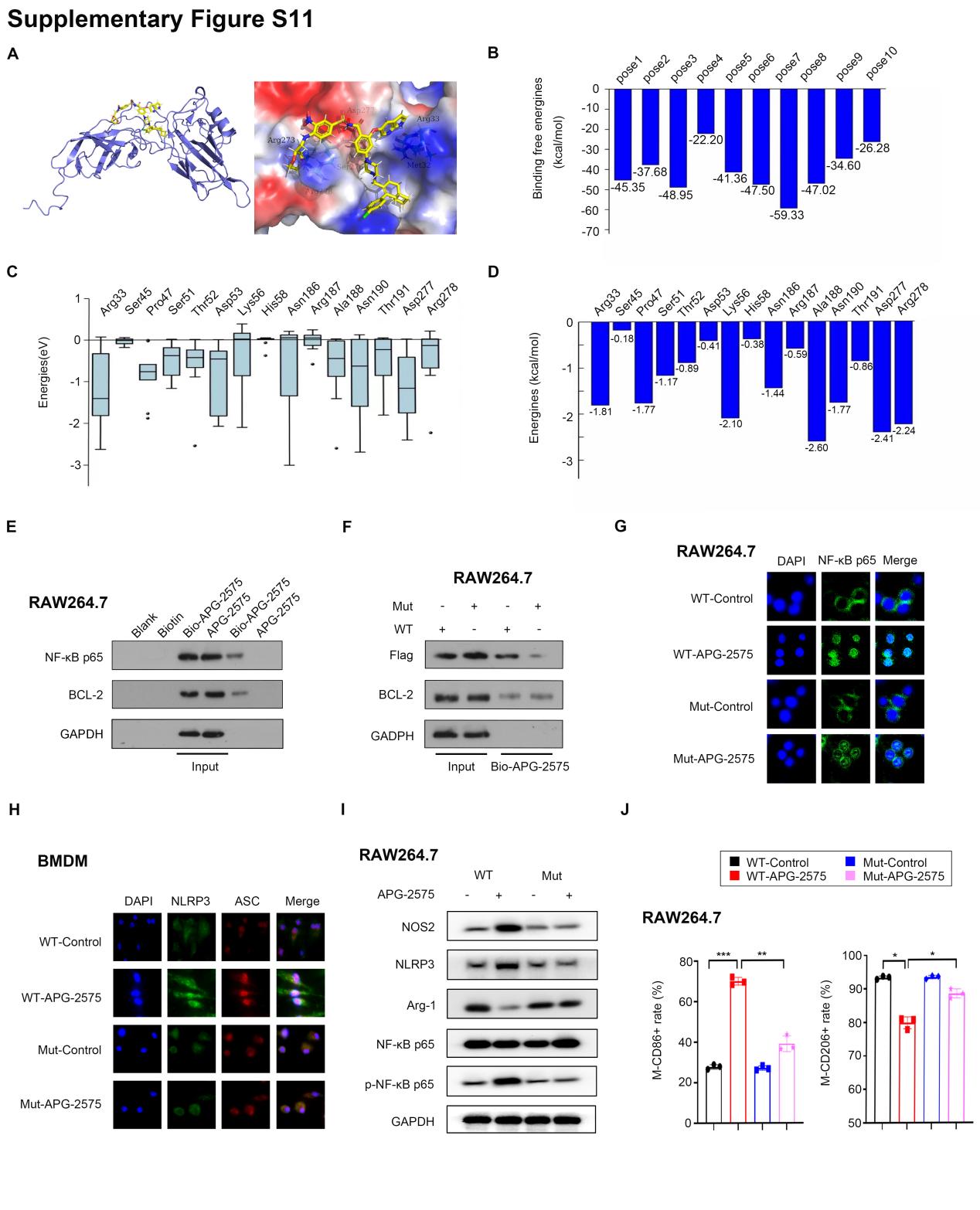
**

**Supplementary Figure S11: APG-2575 induced NLRP3 transcription via enhancing NF-κB nuclear localization.**

(A) The detailed 3D binding mode of mouse RELA (NF-κB p65) with APG-2575. (B) The binding free energies of the top ten scored poses for mouse RELA protein. (C) Box plots of the per-residue energy decomposition (PRED) were within 4.0 Å between APG-2575 and mouse RELA protein for all poses. (D) PRED of residues within 4.0 Å between APG-2575 and RELA protein for the lowest energy poses. (E) Bio-APG-2575 was added to streptavidin-agarose beads and incubated. Biotin alone was used as a control. Lysates prepared from RAW264.7 cells. (F) RAW264.7 cells were transfected with WT (wild type) NF-κB p65 or mutant NF-κB p65 (Arg33A/Lys56A/Asp277A/Arg278A). Lysates were added to pull-down assays to detect APG-2575 binding using pull-down assay described in E. (G) NF-κB p65 in IL-4-activated RAW264.7 transfected with WT NF-κB p65 or mutant NF-κB p65 with or without APG-2575 treatment as examined using a confocal fluorescent microscope. Green, NF-κB p65; blue, DAPI. Scale bar, 20 μm. (H) Representative immunofluorescence staining of NLRP3, ASC, and DAPI on IL-4-activated BMDM transfected with WT NF-κB p65 or mutant NF-κB p65 with or without APG-2575 treatment as examined using a confocal fluorescent microscope. Scale bar, 20 μm. (I) Western blotting analysis of NOS2, NLRP3, Arg-1, NF-κB p65 of IL-4-activated RAW264.7 transfected with WT NF-κB p65 or mutant NF-κB p65 with or without APG-2575 treatment. (J) Flow cytometry analysis of CD86 and CD206 of IL-4-activated RAW264.7 transfected with WT NF-κB p65 or mutant NF-κB p65 with or without APG-2575 treatment.
